# Establishing an Elastography calibration standard: Validation of a shear wave TOF device for measuring Elasticity and Viscosity in tissue-mimicking phantoms using rheometry

**DOI:** 10.1371/journal.pone.0335645

**Published:** 2025-11-13

**Authors:** Jotham Josephat Kimondo, Yi Hu, Junjie Xue, Bangyi Luo, Ziang Feng, Jun Wu, Zhe Wu

**Affiliations:** 1 School of Life Science and Technology, University of Electronic Science and Technology of China, Chengdu, China; 2 School of Medical Imaging, North Sichuan Medical College, Nanchong, Sichuan, China; 3 Tianfu Jincheng laboratory, City of Future Medicine, Chengdu, China; King Abdulaziz University, SAUDI ARABIA

## Abstract

This study designed a novel shear wave Time of Flight (TOF) device to measure frequency-dependent shear wave velocity in tissue-mimicking materials, from which viscoelastic parameters were estimated through Kelvin-Voigt fractional derivative modeling to establish a reliable calibration standard. Tissue-mimicking phantoms were fabricated using 10 wt% polyvinyl alcohol (PVA) and 2 wt% α-alumina powder, with mechanical properties modulated through freeze-thaw cycling. Bimorph transducers operating in the 40–180 Hz range induced and captured shear waves. A single-cycle sine wave excitation ensures narrowband propagation, and a custom algorithm based on the cumulative energy technique robustly detects the shear wave arrival time to estimate TOF. Frequency-dependent shear velocity data were fitted to the Kelvin Voigt fractional derivative (KVFD) model to derive the relaxed elastic modulus (*E*_*o*_), viscosity (η), and fractional order (α), with Poisson’s ratio and damping effects accounted for in the model assumptions. The fitting demonstrated high accuracy, with an R^²^ value of 98.8% (RMSE = 0.013 m/s) for the hard phantom and 99.1% (RMSE = 0.002 m/s) for the soft phantom. Validation with standard rheometer data showed reasonable agreement in elasticity, with percent differences of 2.1% for the hard and 13.3% for the soft phantoms. The latter reflects greater sensitivity to damping effects and assumptions on Poisson’s ratio, as reported in previous studies. However, η and α showed larger deviations because they are strongly dependent on the measurement band; therefore, a direct comparison of these parameters across techniques with nonoverlapping frequency ranges is inappropriate. To enable a fair cross-method assessment, we performed band-matched velocity domain projections in both directions using the KVFD forward model and a constrained TOF refit with *E*_*o*_ fixed to the rheometer value. This analysis revealed that the discrepancies in *η* and *α* primarily stem from frequency band sensitivity rather than methodological bias. These findings support the shear wave TOF device as a robust, frequency-tunable alternative to rheometry for ex vivo tissue characterization and for calibrating clinical elastography. Its immediate clinical relevance is to provide a rapid and low-cost approach for phantom standardization and to inform elastography parameter settings. Key limitations of the present study are the restriction to ex vivo validation, operation within 40–180 Hz, and use of a dispersion-only inversion model; consequently, the viscous parameters *(η, α)* are frequency sensitive and not directly comparable to low-frequency rheometry. Future evaluation of in vivo performance and spatial heterogeneity is therefore essential.

## 1. Introduction

Recent advancements in medical research have increasingly emphasized the importance of biomechanical properties in understanding tissue behavior and pathology. Elasticity and viscosity of biological tissue have emerged as critical parameters, demonstrating significant potential in medical applications, particularly in disease diagnosis and monitoring of treatment outcomes [1–4]. Scientists revealed the viscoelastic properties of biological tissues associated with inflammation levels, a crucial factor in the progression of diseases such as cancer, nonalcoholic fatty liver disease (NAFLD), fibrosis, and nonalcoholic steatohepatitis (NASH) within tissues like the breast, liver, prostate, and plantar [2,3,5–7]. By understanding these variations in the biomechanical properties of biological tissues, researchers aim to develop effective strategies for early intervention, ultimately enhancing patient care and outcomes. Studies have highlighted various technological innovations that researchers developed and utilized to investigate these diseases within biological tissues by characterizing the viscoelastic properties of these tissues, either qualitatively or quantitatively. Among existing methods used to measure the elasticity of biological tissues qualitatively are strain elastography (SE), which employs manual compression techniques to observe the tissue response in terms of strain, and Acoustic radiation force impulse (ARFI) imaging, which generates localized tissue displacements using focused ultrasound pulses [8–11]. The resulting tissue deformation is analyzed to infer stiffness, with stiffer tissues, such as tumors or fibrotic areas, exhibiting less deformation under compression compared to softer tissues. SE and ARFI imaging generate a color-coded elastogram, superimposed on the ultrasound image, where stiffer tissues typically appear in shades of blue.

In comparison, red or green represented softer tissues. Unlike qualitative methods, researchers employ quantitative approaches to calculate numerical values of viscoelastic properties and generate quantitative maps of stiffness, as well as, more recently, viscosity imaging [8,9,11]. Among the existing techniques is Shear wave elastography (SWE), which utilizes acoustic radiation force to induce shear wave propagation, a significant parameter related to the viscoelastic properties of biological tissues. Additionally, Transient Elastography (TE) and Magnetic Resonance Elastography (MRE) utilize controlled external excitation to propagate shear waves through the tissue [12–15]. Other techniques that quantify viscoelastic properties and are considered gold standards, despite their limitations in clinical settings, include uniaxial mechanical tensile and compression tests, Dynamic mechanical analysis (DMA), and rheometry [10,16]. These techniques rely on applying controlled stress or strain to the material and measuring the resulting deformation or flow behavior. Specifically, uniaxial tests measure the material’s response to static tensile or compressive loads, while DMA assesses the material’s viscoelastic response under oscillatory stress. Conversely, rheometry evaluates the flow properties of materials under shear stress [16].

While established methods, such as strain elastography and dynamic elastography (DE), including SWE, VCTE, SDUV, and bench techniques like DMA and rotational rheometry, have advanced our understanding of material characterization, each presents limitations for ex vivo soft tissues. Strain elastography lacks standardization and is operator-dependent, as estimates vary with the applied compression. DE methods are strongly affected by rapid shear wave amplitude decay due to the media and geometry; in practice, their usable bandwidth is often constrained at higher frequencies, making dispersion challenging to track under attenuation. DMA and rheometry emphasize low frequencies, require sample preparation that can disturb the structure, and involve complex systems that necessitate expertise. Across elasticity measurements, however, time-of-flight (TOF) is a common backbone in ultrasonics, in which echo TOF gives speed of sound and thickness through **c = 2D/*Δ*t,** in SWE and VCTE, lateral wavefront arrival times provide *c*_*s *_*= D/Δt*, in SDUV, phase delay slopes across distance and frequency yield group delay and dispersion. Building on this paradigm, we implement a device with a short-path shear wave Time-of-Flight (TOF) protocol that measures frequency-dependent shear wave velocity from travel time differences across a known distance D. By contrast with other methods of TOF estimation, in our study, we extract TOF using the cumulative energy of both transmitted and received signals. The knee point, identified through the derivative of the cumulative energy, marks the onset of the shear wave at both the transmitter and receiver. The difference in this onset point is that it gives a TOF. This onset-based pick mitigates cycle ambiguity and is more robust to attenuation and multi-cycle receiver waveforms than simple thresholding or cross-correlation. Compared with DMA and rheometry, TOF-based shear wave measurements operate over a practical frequency range on small, intact samples with minimal preparation, utilizing compact, low-cost instrumentation with a frequency-tunable band (here, 40–180 Hz) that probes dispersion. Immediate use cases include cross-method calibration of elastography phantoms and rapid ex vivo characterization of surgical or biobank specimens, including small biopsies. Because it directly yields speed, we deduce viscoelastic parameters by fitting the observed dispersion to a Kelvin-Voigt fractional derivative (KVFD) model formulated in the complex shear modulus. The forward model is loss-aware, and a separate attenuation fit is unnecessary. In heterogeneous media, the measured *c*_*s*_ is a path-averaged property; accuracy is best when the dominant heterogeneity scales exceed the wavelength of interest. We mitigated confounds by enforcing far-field geometry **(D ≥ 2*λ)*, maintaining greater than one-wavelength boundary clearance, and retaining only adequate SNR frequencies. The following section reviews existing methods (SWE, VCTE, SDUV, DMA, rheometry) to situate this TOF-based framework in the broader context.

### 1.1 Overview of existing methods

***Shear wave elastography (SWE):*** Shear wave elastography (SWE) techniques, including Point shear wave elastography (P-SWE) and 2D-Shear wave elastography (2D-SWE), are widely used for assessing tissue elasticity [15]. These techniques generate shear waves by focusing ultrasound pulses within the region of interest (ROI) and using ultrasound to track the propagation velocity of these waves. SWE measures *c*_*s*_ and constrains **G = *ρ*c**_*s*_^*2,*^ where *ρ* is tissue density and *c*_*s*_ is shear wave speed. It reports *E* through **E = 2(1 + *ν*) G,** so **E = 3*ρ*c**_*s*_^*2*^ only when *(ν ≈* 0.5)**. In viscoelastic media, G*(ω) is complex; we therefore model in G* and convert *E* for reporting with *ν* specified [17,18]. Studies, such as those conducted by [12,13], have demonstrated the efficacy of SWE in characterizing the advanced stages of various diseases. In liver tissue, for instance, SWE has emerged as a key tool for assessing the extent of fibrosis in patients with chronic viral hepatitis [13,13,19]. Further research has proposed cut-off values to differentiate between malignant and benign breast lesions using SWE [20]. However, challenges remain in justifying these findings, as earlier clinical studies have indicated that factors like tissue viscoelasticity, nonlinearity, and anisotropy influence the interpretation of shear wave speed (SWS) and the estimation of the elastic modulus [21,22]. Consequently, researchers are increasingly interested in assessing viscosity as a critical parameter alongside elasticity, recognizing that viscosity plays a significant role in the nonlinearity and heterogeneity of tissues, particularly during disease progression [21,18].

***Vibration-controlled transient elastography (VCTE)*** uses a similar method to SWE. Still, the primary difference is that VCTE utilizes an external vibrator to generate shear waves systematically, providing a controlled attenuation parameter (CAP) associated with steatosis measurements. Steatosis (Fat accumulation in the liver) alters the liver microstructure, making the tissue more heterogeneous, which may potentially influence changes in the liver’s viscosity. CAP calculates the attenuation by analyzing the decrease in ultrasound signal amplitude as it propagates through the liver [23]. Findings from [24] demonstrated the promising results of the VCTE technique in grading steatosis. The Fibro Scan 502 touch (Echosens, Paris, France) system, deploying the VCTE technique, is recognized as a standard noninvasive technique for detecting fatty liver; however, this method can be susceptible to errors due to beam divergence that occurs when ultrasound propagates through the liver, leading to a decrease in signal amplitude that is unrelated to fat content [21,25,14]. Moreover, clinical studies [23,26,27] have identified BMI, fibrosis, and cholestasis as key factors that contribute to measurement discrepancies and hinder the differentiation of adjacent stages of steatosis.

***Shear wave dispersion ultrasound vibrometry (SDUV)*** is an advanced ultrasound-based technique that provides insights into the elastic and viscous components of biological tissues. SDUV generates the shear waves using a focused ultrasound beam and evaluates their dispersion characteristics. Studies like [20,21] have particularly demonstrated the effect of tissue’s mechanical properties on wave speed and the frequency-dependent variation in shear velocity. Using this dispersion data, [20] showed how significant SDUV can quantify tissue viscoelastic properties. Studies by Shigao Chen et al. [19] demonstrated that SDUV-derived viscosity and elasticity estimates correlate with the degree of fibrosis. Visit Kumar [20] found significant optimal viscosity values that differentiate normal from benign and malignant breast masses. Despite the effectiveness of this method, a discrepancy remains in the findings regarding the estimated viscosity parameter from SDUV [13]. Therefore, the following scientific perspectives are considered a cause of the discrepancy. Firstly, SDUV operates only within the frequency range of 50 Hz-400 Hz, which means that SDUV cannot capture the shear wave dispersion due to attenuation in highly dispersive tissues. Secondly, most studies have quantified mechanical parameters using a linear mathematical model that does not accurately reflect the biomechanical properties of tissues [28].

***Other techniques***: Researchers typically use uniaxial tensile and compression tests, Dynamic mechanical analysis (DMA), and rheometry as reference standard methods for material characterization, depending on the material being studied [3,7,16]. Ying Zhu [29] validated the impact of viscosity on the elasticity results of the gelatin phantoms from SDUV and ARFI using DMA tests conducted with a rotary rheometer (AR1000, TA Instruments, New Castle, DE, USA). Xiao Chen [10] presented a method that quantifies elasticity and viscosity based on laser speckle contrast imaging (LSCI) and validated their method using a conventional rheometer (Discovery HR-2, TA Instruments, USA). While these methods offer unique advantages, their main limitation is the assumption of isotropy, which prevents them from adequately capturing anisotropic stress states or heterogeneity in biological tissue, often resulting in measurement inaccuracies [16]. DMA’s assumption of linear viscoelasticity may not hold at high strains or frequencies, where nonlinear effects become significant [30]. Moreover, rheological measurements are challenging to perform in clinical settings for soft tissue characterization due to the following reasons. Firstly, it isn’t easy [7]. Secondly, the complexity of the equipment makes it very costly and requires high-level expertise to operate. Thirdly, rheometer measurements could not capture mechanical properties for highly dispersive biological tissue, as the device is limited to a frequency range (0.01 Hz- 100 Hz) [31,32].

### 1.3 Research gap

Although uniaxial mechanical tensile and compression testing, DMA, and rotational rheometry are widely used references [30,33], they have significant limitations for ex vivo biological tissue measurements, as outlined in the introduction. To address these gaps, we developed a standard, traceable device that combines a short-path TOF protocol with the KVFD model to fit shear wave dispersion data, enabling a comprehensive characterization of soft-tissue mechanics and supporting cross-method calibration. This study aims to design and evaluate this TOF-based instrument for quantifying tissue elasticity and viscosity across representative tissue types.

### 1.4 Background and modeling framework

To develop a calibration device that quantifies viscoelastic parameters of various tissue materials, we draw upon the principles of viscoelasticity theory, implemented through the KVFD model, and use TOF as a key measurement method.

#### Viscoelastic model.

Biological tissues are a viscoelastic medium. We characterized its mechanical behavior under dynamic loading by a complex modulus E*(ω) or G*(ω) in shear, decomposed into storage modulus **E’(*ω)* (real part) that describes the elastic or energy-storing component, and the loss modulus **E’‘(*ω)* (imaginary part) that defines the viscous or energy-dissipating component. This frequency-dependent viscous component gives rise to mechanical wave dispersion, meaning the shear wave velocity *c*_*s*_*(ω)* and attenuation *α*_*att*_ *(ω)* vary with frequency through their link to G*(ω) [32,34]. Throughout this paper, ‘dispersion’ denotes the frequency dependence of the shear wave velocity *c*_*s*_*(ω)*, which we estimate through the TOF-based device. Previous studies have modeled tissue viscoelasticity using classical linear models, such as the Kelvin-Voigt (KV), Maxwell, and Standard Linear Solid (Zener) model [16,33] s. While useful, these models have inherent limitations for modeling biological tissue. Specifically, they are characterized by a discrete and limited number of relaxation times, which often results in an inability to accurately capture the broad-spectrum, frequency-dependent behavior of tissues across a wide bandwidth [15]. To address this, we employ the KVFD model. The KVFD model is governed by a power-law relaxation spectrum, which allows it to capture a continuous distribution of relaxation times and accurately model the substantial dispersion of shear waves observed in soft tissues over a wide frequency range (1 Hz – 1 kHz) [35]. Capturing power law rheology is critical in medical applications because inflamed tissues often exhibit a nonlinear [16], power-law mechanical response to deformation [36]. The inflammatory process alters tissue microstructure and fluid dynamics, increasing viscosity and enhancing these power-law dynamics [14,37]. The KVFD model incorporates this behavior through a key parameter, the fractional order (α) [36]. This fractional order does not merely assert “viscoelastic,” which all classical models possess. Instead, it controls the frequency dependence of storage and loss over a wide band, enabling a realistic power-law response in soft tissues [15,32].

The KVFD model includes a Hookean spring in parallel with a fractional dashpot, often referred to as a parabolic dashpot to describe its intermediate behavior between an ideal spring and an ideal dashpot. In this setup, the spring pot’s stress is proportional to the fractional time derivative of strain of order **(0 < *α* < 1),** which yields power-law creep and a frequency-dependent loss consistent with soft tissue. Physically, α interpolates between an ideal spring *(α →* 0)** and a Newtonian spring *(α →* 1)**, providing an intermediate, memory-bearing response that efficiently captures broadband dispersion and attenuation. This viscoelastic model has three parameters: *E*_*0*_*, η*, and *α*. *E*_*0*_ represents the relaxed elastic constant, *η* denotes the viscosity parameter, and *α* indicates the order of the fractional derivative. The relationship between stress and strain in the KVFD model is expressed as a constitutive differential equation in the time domain.


$$\sigma (t)=E_{\text{0}}\epsilon (t)+\eta D^{\alpha }[\epsilon (t)]$$ (1)

Where *σ* is the stress, *Ɛ* is the strain, and ‘*t’* is time.

Taking the Fourier transform of equation 1 yields the constitutive equation of the KVFD model in the frequency domain.


$$\sigma (\omega )=E_{\text{0}}\varepsilon (\omega )+\eta (j\omega {)}^{\alpha }\varepsilon (\omega )$$ (2)

Upon simplification, this model yields a complex Young’s modulus, E*(ω). The real and imaginary components of E*(ω) represent the frequency-dependent storage and loss moduli of the viscoelastic material, respectively.


$$E^{*}(\omega )=\frac{\sigma (\omega )}{\varepsilon (\omega )}=E_{\text{0}}+\eta e^{j\frac{\pi \alpha }{\text{2}}}\omega^{\alpha }$$ (3)

Where ω is the angular frequency and E*(ω) is the complex Young’s modulus. Since *ω = 2πf*, we can express the complex Young’s modulus as a function of frequency.


$$E^{*}(f)=\left[E_{\text{0}}+\eta \text{cos}\left(\frac{\pi \alpha }{\text{2}}\right)(\text{2}\pi f{)}^{\alpha }\right]+j[\eta \text{sin}\left(\frac{\pi \alpha }{\text{2}}\right)(\text{2}\pi f{)}^{\alpha }]$$ (4)

The magnitude of E*(f) is obtained as;


$$\left|E^{*}(f)\right|=\;\sqrt{E_{o}^{\text{2}}+\text{2}E_{o}\eta \text{cos}\left(\frac{\pi \alpha }{\text{2}}\right)(\text{2}\pi f{)}^{\alpha }+\eta^{\text{2}}(\text{2}\pi f{)}^{\text{2}\alpha }}$$ (5)

Under harmonic shear excitation, a complex wavenumber-governed wave propagation in a linear, isotropic, viscoelastic medium like biological tissues, as;


$$k(\omega )=\beta (\omega )-i\alpha_{att}(\omega )=\omega \sqrt{\frac{\rho }{G^{*}(\omega )}}=\omega \sqrt{\frac{\text{3}\rho }{E^{*}(\omega )}}$$ (6)

Where ρ is density, G*(ω) and E*(ω) are the complex shear and Young’s moduli, and E* = 2(1 + ν) G*. Equation (6) follows from the viscoelastic wave equation and is standard in elastography; a detailed derivation in terms of E*, |E*|, and real part *E *′ =ℜ{E*} appears in Parker’s and Zvietcovich [38,39].

We write for phase velocity *c*_*s*_*(ω)* and attenuation *α*_*att*_*(ω*),** $E^{*}(\omega )=\;\left|E^{*}(\omega )\right|e^{i\delta_{E}(\omega )}$ with loss angle $\delta_{E}=argE^{*}$. Separating the real and imaginary parts of *k* yields


$$C_{S}(\omega )=\;\frac{\omega }{\beta (\omega )}=\frac{\sqrt{\frac{\left|E^{*}(\omega )\right|}{\left(\text{3}\rho \right)}}}{\text{cos}\left(\frac{\delta_{E}(\omega )}{\text{2}}\right)}$$ (7)

$$\alpha_{att}(\omega )=\;\omega \sqrt{\frac{\text{3}\rho }{\left|E^{*}(\omega )\right|}}\;\text{sin}\left(\frac{\delta_{E}(\omega )}{\text{2}}\right)$$ (8)

These are algebraically equivalent to the expressions given in Parker’s [38]; Zvietcovich et al. provide the companion form in terms of |E*|, *E′* [39].

We use the KVFD constitutive law as shown in equation (4), from which we get the storage modulus and loss modulus as shown in equation (9),


$$E^{\prime }(f)=\;E_{O}+\;\eta (\text{2}\pi f{)}^{\alpha }\text{cos}\left(\frac{\pi \alpha }{\text{2}}\right),\;E^{\prime \prime }(f)=\;\eta (\text{2}\pi f{)}^{\alpha }\text{sin}\left(\frac{\pi \alpha }{\text{2}}\right)$$ (9)

with magnitude


$$\left|E^{*}(f)\right|=\;\sqrt{E^{\prime }(f{)}^{\text{2}}+{\text{E}}^{\prime \prime }(f{)}^{\text{2}}}$$ (10)

Expanding the half-angle in [7] using $\text{cos}\left(\delta_{E}\right)=\;E^{\prime }/\left|E^{*}\right|$, gives a compact, loss-aware expression that depends only on $E^{\prime }(f)$ E″(f)


$$C_{s}(f)=\;\sqrt{\frac{\text{2}\left(E^{\prime }(f{)}^{\text{2}}+E^{\prime \prime }(f{)}^{\text{2}}\right)}{\text{3}\rho \left(\sqrt{E^{\prime }(f{)}^{\text{2}}+E^{\prime \prime }(f{)}^{\text{2}}}+E^{\prime }(f)\right)}}$$ (11)

and, for completeness,


$$\alpha_{att}(f)=\;\omega \sqrt{\frac{\text{3}\rho }{\text{2}}}\sqrt{\frac{\left|E^{*}(f)\right|-E^{\prime }(f)}{{\left|E^{*}(f)\right|}^{\text{2}}}}$$ (12)

The forms follow Parker’s general treatment of complex wavenumbers and align with the |E*|/E *′* decomposition introduced by Zvietcovich et al.

Finally, we relate the complex Young’s modulus E* and the complex shear modulus G* through Poisson’s ratio (*ν*), according to E* = 2(1 + ν) G*. Since soft tissues are often modeled as nearly incompressible (*ν* ≈ 0.5) [40–42], this reduces to the widely used approximation E* ≈ 3 G* in elastography [41]. However, it is essential to note that *ν* may vary depending on tissue type and pathological condition. For comparability with elastography practice, we fix *ν = 0.5* and state this assumption explicitly. Because *ν* may vary across tissues and conditions, we assessed v-sensitivity around *ν = 0.5* by perturbing ν by ±0.01, ± 0.02, and ±0.05 changes, which resulted in approximately 0.7%, 1.3%, and 3.4% changes in the reported E, respectively. At the same time, dispersion trends and goodness of fit are unchanged. Thus, within reasonable ranges, the *ν* assumption induces only small percentage shifts in *E* and does not affect our conclusions. We note this assumption when comparing against rheometry. Building on these established formulations, we express the lossy wave equation regarding the KVFD model, which enables us to derive a compact form of shear wave velocity c_*s*_*(f)* (equation 11) directly in terms of *E*_*o*_*, η,* and *α* parameters we aim to quantify.

## 2. Materials and methods

### 2.1 Conceptual framework

**Fig 1:**
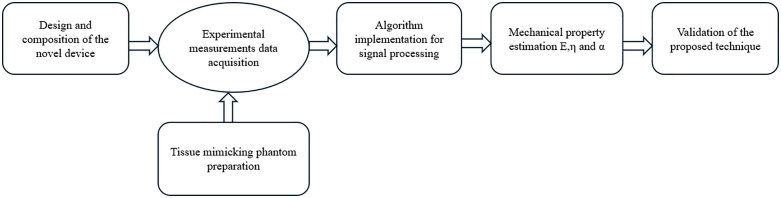
Proposed conceptual framework.

The proposed methodology was structured into five key steps, as outlined in the flowchart presented in Fig 1:

### 2.2 Design and composition of the novel device

**Fig 2:**
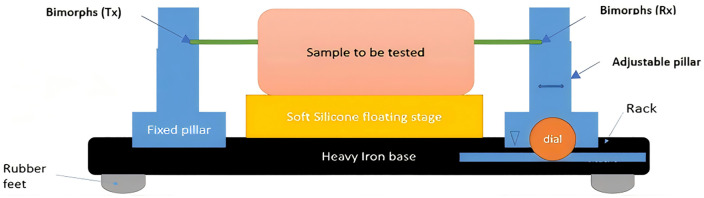
Building block of a proposed equipment.

The proposed device consists of four primary components: a signal generator (DDS), a microcontroller unit, a linear amplifier, and a digital oscilloscope for data acquisition, along with two bimorph sensors operating within a bandwidth of 0–1000 Hz, capable of converting electrical energy into mechanical deflection through the inverse piezoelectric effect. One bimorph serves as the transmitter, while the other functions as the receiver. These bimorphs were positioned at a fixed distance apart, with the tissue-mimicking phantom placed between them, as illustrated in Fig 2. The transmitting bimorph generates vibrations in response to electrical excitation provided by the signal generator. These vibrations generate shear waves in the tissue, mimicking a phantom, which propagate through the medium and reach the receiving bimorph. The receiving bimorph converts the mechanical motion of the shear waves into an electrical signal. The microcontroller unit precisely controls critical parameters such as frequency, voltage, and pulse delay from the signal generator.

The physical setup of the device includes a heavy iron base with a rubber layer positioned beneath it to dampen vibrations. Fixed pillars are attached to the top of the base on both sides, and the transmitting and receiving bimorphs are securely mounted onto these pillars using non-conducting screws. We implemented this design to minimize shear wave propagation through the base or underlying structure, ensuring accurate wave propagation measurement within the phantom.

### 2.3 Fabrication of tissue-mimicking phantoms

We fabricated soft and hard tissue-mimicking phantoms from polyvinyl alcohol- PVA (99% hydrolyzed, molecular weight 89,000–98,000, Sigma Aldrich, St. Louis, MO, USA) and high-purity α-alumina powder (mean particle size ≈ 1 µm, ≥ 99% purity, Sigma Aldrich 1344-28-1) following the thaw protocol described previously in [43–45]. We structured the phantoms in cylindrical shapes with dimensions of 30 mm in diameter and 60 mm in height. We selected PVA cryogels for their tunable elasticity, thermal stability, and durability. At the same time, α-alumina ensures uniform dispersion and enhanced acoustic scattering without compromising mechanical integrity.

#### Preparation *of* PVA solution.

In a conical flask, 100 mL of distilled water was poured, and 10g of PVA powder was gradually added for over 30 minutes while stirring continuously at 1500 rpm. Once the polymer had fully dissolved, 2g (2 wt% %) of high-purity α-alumina powder (mean particle size ≈ 1 µm, ≥ 99% purity, Sigma Aldrich 1344-28-1) was slowly dispersed into the mixture as an acoustic scatter phase. The suspension was stirred for an additional 90 minutes to yield a homogeneous, bubble-free gel solution.

#### Molding and freeze-thaw cycles.

**Fig 3:**
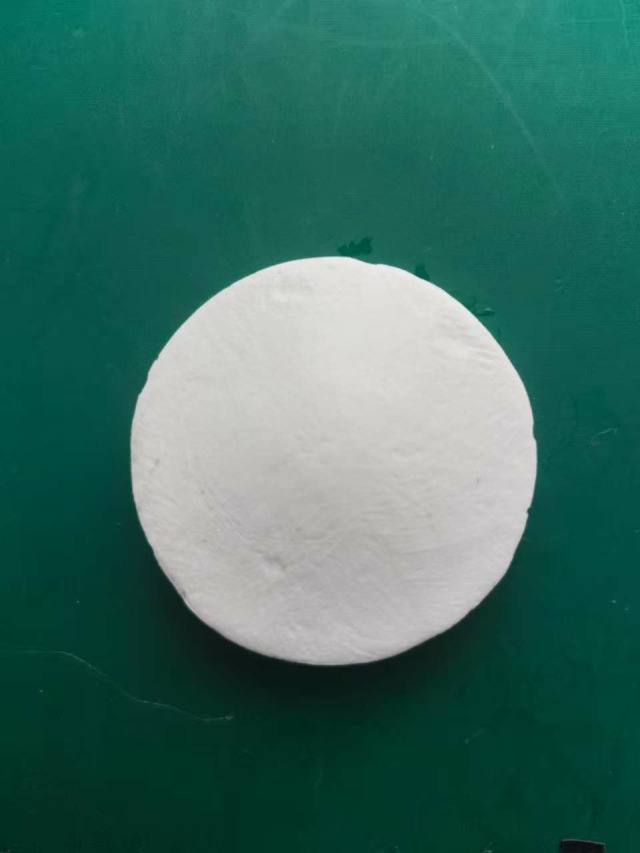
Fabricated PVA-based phantom with identical geometry used for both soft and hard tissue mimics; mechanical properties were varied by adjusting freeze-thaw cycles.

Briefly, a 10% w/v PVA solution was cast into cylindrical molds and subjected to freeze–thaw cycles (−20 °C for 12 h, + 20 °C for 12 h). Following the observations made by Natalia Arteaga-Marrero [44]. The strength and elasticity of the phantoms were primarily governed by the concentration of PVA and the number of freeze-thaw cycles necessary to crosslink the polymer. In this study, we optimized the number of freeze-thaw cycles to achieve the desired mechanical stability and final stiffness for each phantom type. Hard phantoms underwent five cycles to promote extensive polymer crosslinking, ensuring structural integrity and a high shear modulus. Soft phantoms underwent two cycles to limit crosslinking density, thereby achieving a lower, more bio-relevant shear modulus while maintaining sufficient structural cohesion for handling and testing. After fabrication, the resulting phantoms as shown in Fig 3, were stored in phosphate-buffered saline at room temperature for 24 hours to equilibrate hydration. Table 1 details the material composition of each phantom.

**Table 1:** Tissue-Mimicking phantom constituents.

Material	Hard phantom composition	Soft phantom composition
Polyvinyl alcohol (PVA)	10% w/v solution of PVA	10% w/v solution of PVA
α-Alumina powder	2% wt% high-purity α-alumina (≈1µm, ≥ 99% purity) uniformly dispersed for acoustic scatter.	2% wt% high-purity α-alumina (≈1µm, ≥ 99% purity) uniformly dispersed for acoustic scatter.
Water	Used as a solvent for PVA (balance to make up the total volume)	Used as a solvent for PVA (balance to make up the total volume)
Freeze-thaw cycles	1-5 cycles for increased stiffness	1-2 cycles for a softer texture

### 2.4 Experimental measurements and data acquisition

Measurements were performed for both phantoms using the proposed device and rheometer as our gold standard. Raw data was acquired from the experimental setup using bimorph sensors and a digital oscilloscope. Each measurement was sampled at a rate of 100 kHz, ensuring high temporal resolution for accurate analysis.

#### Experimental setup.

**Fig 4:**
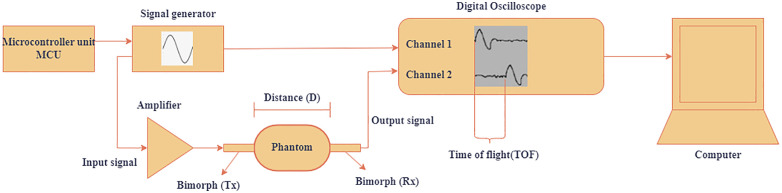
Schematic of experimental setup.

The mechanical properties of soft tissues are closely related to the velocity of wave propagation within the material [46]. In our experimental setup, tissue-mimicking phantoms were positioned between two bimorphs, as illustrated in Fig 4. The transmitting bimorph excited each phantom sequentially using a single sine-burst of frequencies ranging from 40 Hz to 180 Hz in an increment of 20 Hz. The bimorphs were selected for their high resonance frequency and ability to operate in a quasi-static range, enabling our device to function across a broad frequency spectrum. This design allows the characterization of highly dispersive materials.

Although the device can operate within a frequency range of 0 Hz to 1000 Hz, this study focused on a limited range of 40 Hz to 180 Hz to evaluate its performance. We chose 40–180 Hz to balance dispersion leverage with signal fidelity for short-path TOF. The lower bound of 40 Hz ensured stable phase picks, satisfied the far-field criterion **(D ≥ 2*λ)* for all path lengths, and avoided near-field or guided-mode effects that arise at very low frequency, where wavelengths become comparable to or larger than the specimen dimensions and obscure a clear time-of-flight The damping factor, which reduces the received amplitude and dissipates energy, degrades the robustness of our cumulative-energy onset detector and the reliability of phase-slope estimates.. We therefore determined the upper bound of 180 Hz by the onset of significant amplitude decay and SNR loss along the path. Within 40–180 Hz, we consistently observed clean and single-arrival waveforms at the receiver, adequate SNR at both Tx and Rx, and sufficient frequency spread to fit KVFD dispersion robustly while ensuring that our cumulative energy-based onset detection method remained precise and reliable. We excluded frequencies a priori that failed to meet SNR thresholds or violated the condition **(D ≥ 2*λ)*, ensuring that the retained band provided the best compromise between sensitivity to dispersion and resilience to attenuation. The excitation signals were generated using a 5VDC waveform generator (40 Hz-180 Hz) and amplified by a ± 55V linear amplifier before driving the Tx bimorph. Measurements were conducted with an excitation amplitude of 64 V, which induced particle displacement within the phantom fabricated from PVA as it responded to the bimorph’s vibrations. This displacement corresponds to the shear wave motion propagating through the phantom, which was subsequently detected by the receiving bimorph. The system routes the voltage output from the Rx bimorph, which is proportional to local shear displacement, to a 2 GHz digital oscilloscope sampling at 100kHz.The system streams data to MATLAB 2024b (MathWorks, Inc., Natick, MA, USA) for offline processing and TOF estimation.

To determine the shear wave propagation velocity, the distance (D) between the transmitting (Tx) and receiving (Rx) bimorphs must be known precisely. In our setup, both bimorphs are laterally coupled to the side of the phantom using a thin shear coupling layer. This shear polarized geometry excites and senses tangential motion efficiently (S-waves) while coupling poorly to compressional waves. At 40 Hz to 180 Hz, the compressional wavelength is orders of magnitude larger than the sample, so any residual P-wave appears quasi-uniform; our frequency domain phase slope estimate of *c*_*s*_*(f)* rejects it. A constant, low preload was enforced by a mechanical stop integrated into the holder assembly, ensuring identical contact conditions for all experiments by preventing bending of the bimorph elements and minimizing local deformation of the sample. The lateral position of the Rx bimorph is controlled by a micrometer dial integrated into an adjustable pillar (Fig 2), allowing for precise and repeatable adjustments of the internal path length D.

To ensure that the measured wave speed is a property of the material and not influenced by geometric artifacts, we implemented several controls:

1. Far-field criterion: The distance D was chosen to satisfy a far-field condition **(D ≥ 2*λ)* at each excitation frequency to ensure plane wave propagation.
2. Boundary avoidance: A greater than one-wavelength clearance was maintained from all sample edges to minimize reflections and guided mode effects.
3. Invariance validation: To empirically verify that the measured velocity *c*_*s*_ was invariant to the specific measurement geometry, we acquired five measurements per sample, increasing D in 0.5 mm increments for the hard phantom, which was 48.91–51.0 mm, and for the soft phantom, which was 40.56–42.6 mm.

Across these distance settings, the estimated *c*_*s*_ showed no systematic trend and remained within the standard error of the measurements. Therefore, we treat the density (ρ), frequency (f), and path length (D) as known inputs to the model and do not include geometry as a fit parameter. This approach confirms that our results characterize the essential viscoelastic properties of the material. We calculated the shear wave propagation velocity within the phantom using the following relationship:


$$Shear\;velocity=\frac{Distance(D)}{Time\;of\;flight\;of\;the\;shear\;wave}$$

D represents the distance between the edges of the transmitting and receiving bimorphs, and the time of flight corresponds to the time the shear wave travels through the sample.

We connected the terminals of the transmitting bimorph to Channel 1 of a digital oscilloscope and the terminals of the receiving bimorph to Channel 2 to determine the TOF. This setup enabled real-time visualization of the transmitted and received signals, allowing the measurement of the time lag between them. The observed time lag between the pulses on Channel 1 and Channel 2 represents the shear wave propagation time from the transmitting bimorph’s edge, through the sample, to the receiving bimorph’s edge (Fig 4).

### 2.5 Signal processing and noise reduction

**Fig 5:**
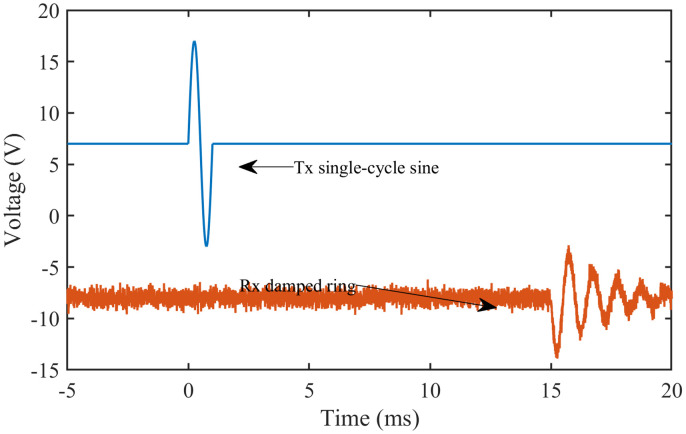
Hypothesized output signal from the receiving bimorph.

Based on the findings of Wu Zhe [47], we hypothesized that the output signal from the receiving bimorph would resemble the waveform illustrated in Fig 5. Consequently, the raw output signal consisted of both white and random noise, influenced by several factors.

The propagating shear waves experienced rapid attenuation due to media dispersion and geometric effects. Secondly, undesired interference signals from multiple sources, including power line noise, electromagnetic coupling, and electronic equipment, were introduced into the output signal as white noise. These factors posed significant challenges in accurately tracking the TOF.

In our setup, the signal recorded on Channel 2 exhibited a null amplitude until the shear wave reached the receiving bimorph (Rx). To track the TOF, we aimed to identify the point in the output signal where the initial signal disturbance occurred. Accurately determining this point required enhancing the signal quality through a structured signal processing protocol.

The processing approach involved the following steps:

1. Multiple signal measurements at fixed time intervals and averaging to reduce random noise and improve signal-to-noise ratio (SNR).
2. A zero-phase band pass FIR filter that avoids phase distortion by forward-backward filtering, designed to sharply attenuate frequencies outside 10–190 Hz.
3. Implementing a median filter to eliminate impulse noise, improve signal clarity, and ensure reliable TOF detection.
4. Wavelet denoising & Savitzky-Golay were applied to remove high-frequency noise and smooth the signal without distorting waveform peaks.

This multi-step filtering strategy enhanced the signal quality, enabling precise identification of the TOF for accurate shear wave velocity calculations. We calculated the SNR before and after filtering to assess the effectiveness of the signal processing steps, as shown in the equation below.


$$SNR=\text{1}\text{0}*{log}_{\text{1}\text{0}}\left(\frac{\left(True\;signal^{\text{2}}\right)}{Filtered\;signal^{\text{2}}}\right)$$ (13)

### 2.6 TOF estimation using cumulative energy and derivatives

The TOF of the shear wave was estimated using cumulative energy analysis. This technique involves the following steps;

1. Hilbert transform to extract analytical signal, envelope detection, and instantaneous power as shown below;

z(t)=x(t)+jH{x(t)} (14)

A(t)=|z(t)| (15)

$$P(t)=A^{\text{2}}(t)$$ (16)

Where z(t) is the analytical signal, x(t) The real signal, A(t), is the envelope of the analytical signal, and P(t) is the instantaneous power of the analytical signal.

2. The cumulative sum of the instantaneous power over time

The objective was to instantaneously detect the onset of disturbance at the point of the received signal. We hypothesized that effective signal processing would allow us to achieve the desired outcome by summing the entire instantaneous power of the signal, as shown in equation 13. At the point of the instantaneous disturbance, which we aim to detect, the cumulative energy curve will exhibit a distinct “knee,” marking the arrival of the shear wave.


$$C(t)=\;\int_{\text{0}}^{t}P(\tau )d\tau$$ (17)

1. Identifying the knee point where the power starts to rise

We calculated the derivative of the cumulative energy to identify the “knee” of the energy curve. The knee point defines the location where the cumulative energy derivative *dC/dt* exceeds the threshold of *µ*_*dC*_* + σdC, where µdC* and *σ*_*dC*_ represent the mean and standard deviation of the signal, respectively. This thresholding technique effectively localizes the arrival of the wave front.

### 2.7 Mechanical property estimation

The dispersion data, representing the shear wave velocity at each excitation frequency, were collected over a range starting from 40 Hz with an increment of 20 Hz. We analyzed this dataset to estimate the mechanical properties of the tested phantoms. We performed curve fitting using MATLAB 2024b (MathWorks, Inc., Natick, MA, USA) on the collected dispersion data. The fitting process aimed to model the frequency-dependent variation of shear velocity, allowing for the extraction of key mechanical parameters, such as elasticity and viscosity, as outlined in Equation 11. We designed the inversion to use dispersion only because the propagation path was short (sample length D < 5 cm), making frequency-dependent amplitude decay along the path negligible *(α*_*att*_*(f) D << 1)* in our band of interest. Consistent with the viscoelastic framework presented in section 1.4, we retained loss awareness in the forward model through the complex modulus and its loss angle (i.e., the damping correction is embedded in the phase-velocity relation); however, we did not fit a separate attenuation spectrum because path length-induced decay was not measurable above noise.

To ensure this choice was appropriate, we: (i) limited the analysis to frequencies with adequate SNR and for which the geometric condition *D ≥ 2λ* held, thereby reducing near-field and guided mode effects; (ii) inspected amplitude spectra across the path and found no systematic frequency-dependent decay over the analyzed band; and (iii) performed a post-fit consistency check showing that the implied loss angle remained modest across the fitted band, indicating that any bias from not fitting an explicit attenuation term would be slight relative to measurement variability.

Parameter estimation utilized weighted least-squares over the selected frequency band, with physically meaningful bounds (i.e., **0 < *α* < 1)**, positive moduli/viscosities, and robust weighting to down-weight low-SNR frequencies. For reporting, we expressed results in Young’s modulus, using the near-incompressibility assumption introduced in section 1.4, with the caution that modest changes in *ν = 0.5* can affect the reported *E* by only a few percent. We evaluated the accuracy of the estimated parameters based on goodness-of-fit metrics, ensuring a reliable characterization of the phantom materials.

### 2.8 Validation of the proposed technique

**Fig 6:**
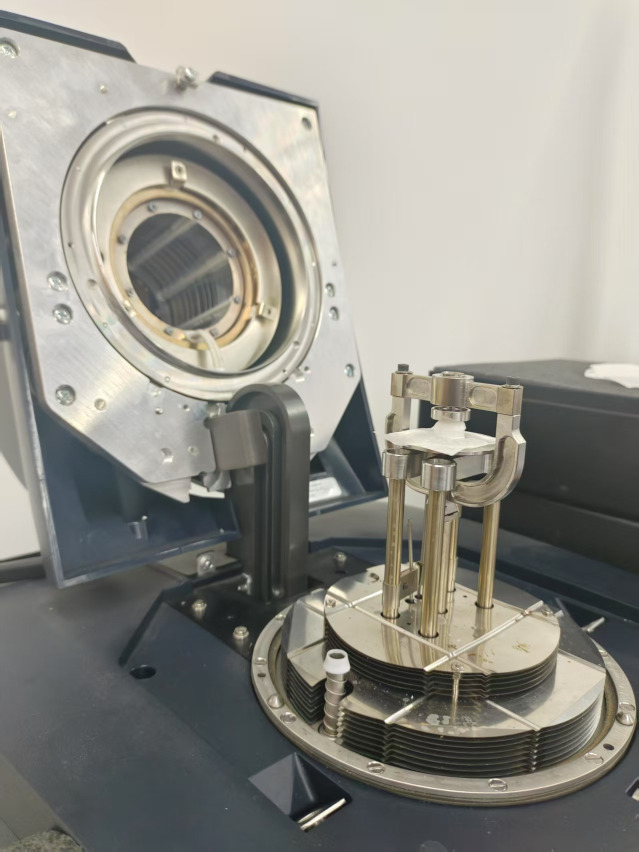
Experimental test setup of the Discovery Series HR-2 rheometer (TA Instruments Inc., New Castle, Delaware, USA) configured for harmonic rotational shear testing using a disc-shaped tissue-mimicking phantom.

To validate the performance of the proposed device, the mechanical properties of the two fabricated phantoms were also measured using a hybrid rheometer as shown in Fig 6, which served as the gold standard for comparison.

The measurements were conducted under identical conditions to ensure consistency and reliability. We performed shear experiments using a parallel plate configuration (15 mm diameter) with the Discovery Series HR-2 rheometer (TA Instruments Inc., New Castle, Delaware, USA). We individually positioned the tissue-mimicking phantoms between the parallel plates of the rheometer, and any excess material at the edges was carefully trimmed using a scalpel to ensure uniform contact. Before conducting the main experiments, we performed strain sweep oscillatory tests to identify the linear viscoelastic region (LVR) across different frequencies. We performed these preliminary tests at 0.1 Hz, 1 Hz, 10 Hz, and 20 Hz, incrementally increasing the strain amplitude from 0.01% to 2%. The results indicated that hard and soft tissue-mimicking phantoms exhibited an LVR at 0.1 Hz and 1 Hz with a maximum strain of 1%, while no LVR appeared at frequencies above 1 Hz. Based on these findings, we selected a strain amplitude of 1% for the subsequent frequency sweep oscillation tests. We performed frequency sweeps within the identified linear regions: from 0.1 Hz to 1 Hz for both hard and soft phantoms, at a constant strain of 1%. For each frequency, the hybrid rheometer provided the storage and loss shear moduli, *G’ (f)* and *G“(f)*. We then converted *G’* and *G”* to shear velocity using the loss-aware formulation in equation 18 [48].

### 2.9 Band-matched comparison across modalities

We conducted band-matched cross-projections in the velocity domain to compare rheometer and TOF-based device estimates fairly across disparate frequency ranges. KVFD parameters **(Eo,* η, α)* were estimated separately in each native band using the KVFD forward model. First, we fitted the model to TOF dispersion measured over the frequency range 40–180 Hz using a loss-aware E-domain forward map from *E’ (f), E“ (f)* to phase velocity as shown in Equation 11. Second, we fitted the same KVFD model to the rheometer dispersion data. For rheometry (0.1–1 Hz), measured *G’ (f)* and *G” (f)* were converted to velocity using the equivalent shear domain formula (equation 18) ensuring the same loss-aware convention.


$$C_{S}\left(G^{\prime },G^{\prime \prime }\right)=\;\sqrt{\frac{\text{2}\left({G^{\prime }}^{\text{2}}+{G^{\prime \prime }}^{\text{2}}\right)}{\rho \;G^{\prime }\left(\text{1}+\sqrt{\text{1}+{\left(G^{\prime \prime }/G^{\prime }\right)}^{\text{2}}}\right)}}\;$$ (18)

We then projected the rheometer fit KVFD parameter to predict *c*_*s*_*(f)* over 40–180 Hz and compared with TOF *c*_*s*_*(f)*, and projected the TOF fit parameter to predict *c*_*s*_*(f)* over (0.1–1 Hz) and compared against rheometer-derived *c*_*s*_*(f)*. Finally, as a physics-guided test, we performed a constrained TOF refit with *E*_*o*_ fixed to the rheometer value while re-optimizing *η and α over the range of* 40–180 Hz.

## 3. Results

### 3.1 Dispersion characteristics

Tables 2–3 summarizes the measured dispersion across the tested path lengths. In both phantoms, the mean shear wave speed increases monotonically with frequency (normal dispersion). The hard phantom rises from 1.39 m/s at 40 Hz to 1.75 m/s at 180 Hz. The soft phantom exhibits the same upward trend, indicating the dispersive nature of tissue-mimicking phantoms. These trends align with the KVFD loss-aware mode used for fitting.

**Table 2:** Summary of the dispersion characteristics of the hard tissue mimicking phantom.

Frequency (Hz)	Velocity1 (m/s)	Velocity2 (m/s)	Velocity3 (m/s)	Mean ±SE
40	1.360	1.390	1.390	1.380 ± 0.010
60	1.413	1.444	1.458	1.438 ± 0.013
80	1.479	1.497	1.501	1.492 ± 0.007
100	1.492	1.505	1.523	1.507 ± 0.009
120	1.513	1.541	1.608	1.5554 ± 0.028
140	1.577	1.603	1.632	1.604 ± 0.016
160	1.660	1.688	1.705	1.684 ± 0.013
180	1.694	1.714	1.750	1.719 ± 0.016

**Table 3:** Summary of the dispersion characteristics of the soft tissue mimicking phantom.

Frequency (Hz)	Velocity1 (m/s)	Velocity2 (m/s)	Velocity3 (m/s)	Velocity4 (m/s)	Velocity5 (m/s)	Mean ±SE
40	0.686	0.691	0.700	0.705	0.711	0.698 ± 0.004
60	0.699	0.709	0.716	0.725	0.727	0.715 ± 0.005
80	0.713	0.724	0.732	0.736	0.744	0.730 ± 0.005
100	0.731	0.733	0.737	0.745	0.761	0.741 ± 0.005
120	0.736	0.739	0.747	0.758	0.763	0.749 ± 0.005
140	0.746	0.745	0.751	0.759	0.767	0.754 ± 0.004
160	0.747	0.754	0.762	0.770	0.778	0.762 ± 0.006

### 3.2 Curve fitting and dispersion data

**Fig 7:**
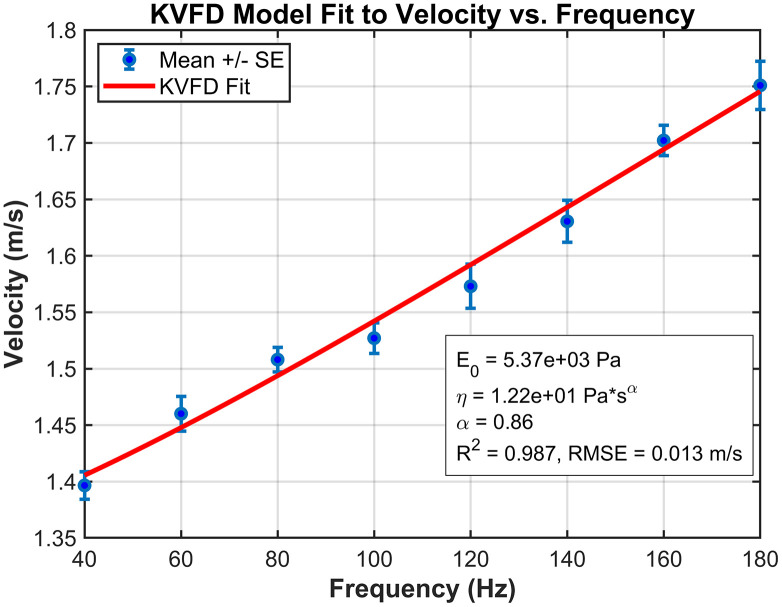
Curve fitting of mean velocity versus frequency for the hard mimicking phantom, comparing experimental data with the theoretical KVFD model.

**Fig 8:**
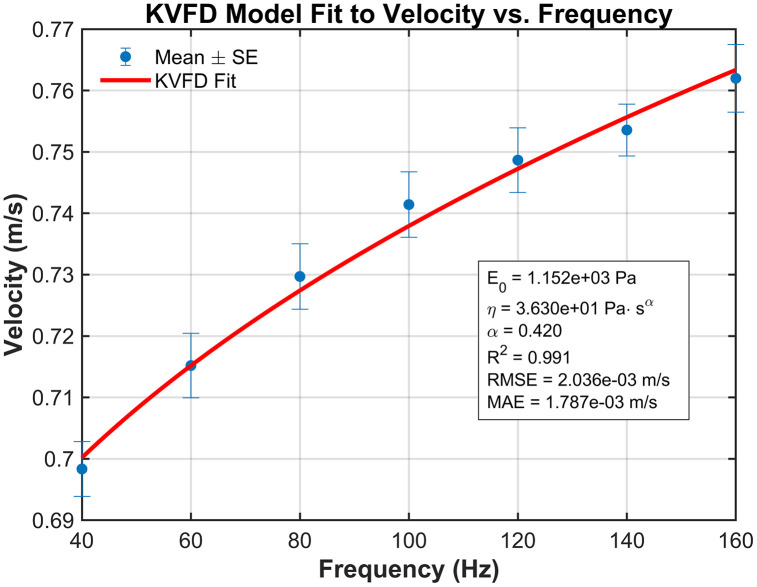
Curve fitting of mean velocity versus frequency for the soft mimicking phantom, comparing experimental data with the theoretical KVFD model.

We applied the KVFD model to the mean shear velocity dispersion data obtained from the TOF device for the experimental setup. Because the TOF approach directly measures shear wave velocity rather than elastic modulus, parameter estimation is performed in the complex Young’s modulus E*(*⍵*). For conversion from the complex shear modulus G*(*⍵*) to elastic modulus, we assumed a Poisson’s ratio of 0.5, consistent with the nearly incompressible behavior of soft tissues as shown in equation 7. Damping effects, which influence wave attenuation, were represented within the KVFD framework through the viscosity (η) and fractional derivative order (α), which capture frequency-dependent energy dissipation together. The curve fitting demonstrated strong agreement with the experimental observations, yielding a coefficient of determination (R²) of 98.7% and a root mean square error (RMSE) of 0.013 m/s for the hard phantom, and an R² of 99.1% with an RMSE of 0.002m/s for the soft phantom. These results, illustrated in Figs 7 and 8, respectively, confirm the suitability of the KVFD model for characterizing the dispersive behavior of both phantom types.

### 3.3 Signal processing results

**Fig 9:**
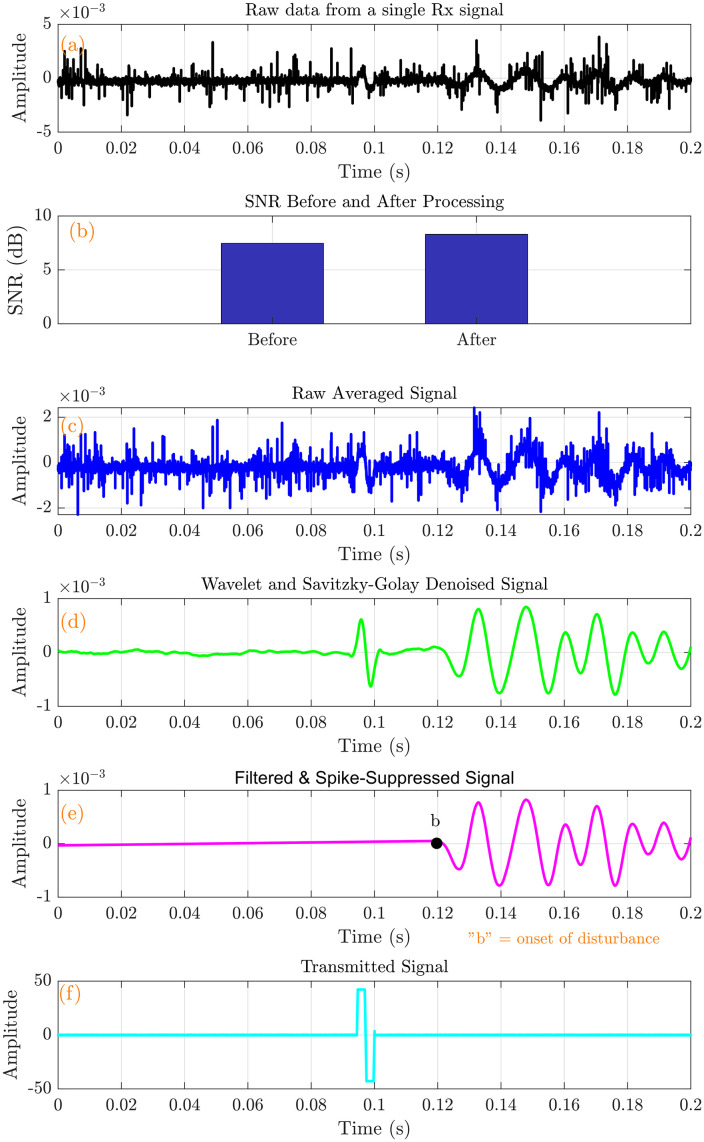
Signal processing pipeline output for the hard tissue mimicking phantom. (a) Raw data from a single Rx signal (b) SNR Improvement (c) Raw average signal (d) Median +Savitzky Golay +Wavelet filtered signal (e) Spike suppressed filtered signal (f) Transmitted signal.

The raw signals were processed using the techniques above, resulting in substantial noise reduction and a marked improvement in signal quality. As shown in Fig 9a, the unprocessed signal exhibited significant noise and distortion. After applying filtering techniques (Fig 9d), the signal became noticeably clearer, with reduced noise and a more distinct identification of TOF. A comparison of the signal-to-noise ratio (SNR) before and after processing revealed a significant enhancement, with an SNR improvement of approximately 0.17dB, also illustrated in Fig 9b.

The denoising procedures, particularly the application of wavelet and Savitzky-Golay filtering, effectively attenuated high-frequency noise components, resulting in smoother and more coherent signals, as shown in Fig 9d. We plotted the transmitted signal (Fig 9f) and the corresponding received signal (Fig 9e) to illustrate the shear wave propagation characteristics further. The observable time delay between these two signals corresponds to the TOF, which is the basis for calculating the shear wave propagation velocity.

### 3.4 TOF estimation results

#### 3.4.1 Cumulative energy and derivatives.

**Fig 10:**
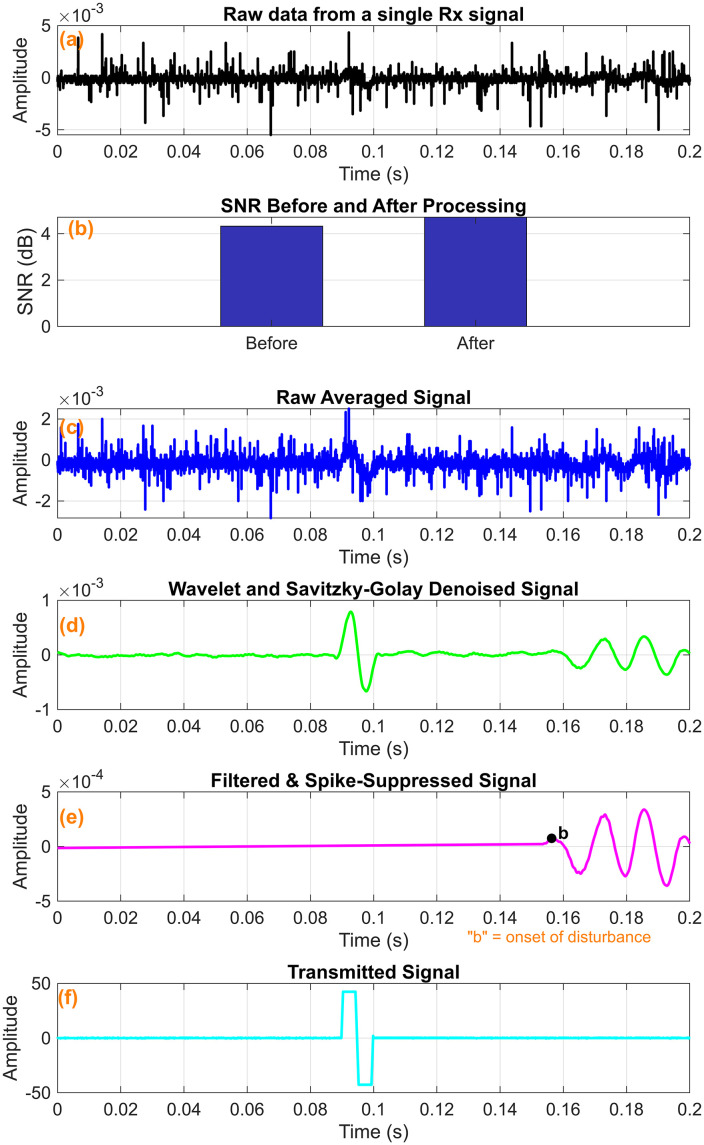
Signal processing pipeline output for the soft tissue mimicking phantom. (a) Raw data from a single Rx signal (b) SNR Improvement (c) Raw average signal (d) Median +Savitzky Golay +Wavelet filtered signal (e) Spike suppressed filtered signal (f) Transmitted signal.

**Fig 11:**
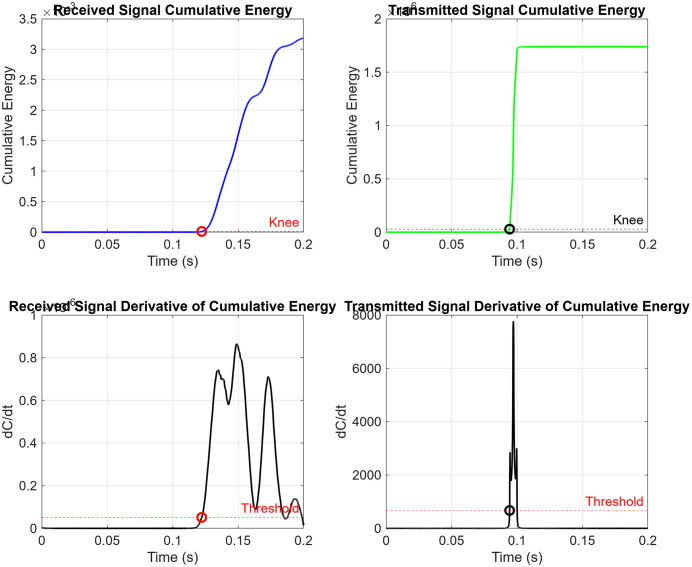
TOF detection via the cumulative energy knee method. (Top) Cumulative energy curves for the transmitted (green) and received(red) signals, with knee points(circles) indicating shear wave arrival times. (Bottom) First derivatives of the cumulative energy curves, with the threshold markers used to localize the knee point corresponding to wavefront onset.

The cumulative energy for both the received signal (Fig 9e) and the transmitted signal (Fig 9f) was plotted as a function of time to illustrate how energy accumulates during shear wave propagation through the tissue-mimicking phantoms (Figs 10,11).

These plots visually represent the energy buildup associated with wave transmission. To further enhance the analysis, we computed the derivatives of the cumulative energy curves, first to accurately identify the optimal threshold at the knee. Secondly, to determine the arrival point of the shear wave, referred to as the “knee point.” This approach enables precise localization of wavefront arrival and offers more profound insight into the propagation dynamics within the medium.

### 3.5 Validation using rheometry

The mechanical properties of the phantoms were validated using a rheometer, which served as the gold standard for comparison. To evaluate the accuracy of the proposed TOF-based method, we first obtained independent measurements of the shear wave time of flight, which yielded frequency-dependent shear velocities. These dispersion curves represent the direct measurements obtained with the TOF device. We derived key viscoelastic parameters, such as relaxed elastic modulus *(E*_*o*_*)*, viscosity *(η)*, and fractional derivative order *(α)*, by fitting the velocity and frequency data to the KVFD model. By distinguishing between directly measured quantities and derived parameters, we can achieve conceptual rigor and clarify the basis for comparing TOF with rheometry.

**Fig 12:**
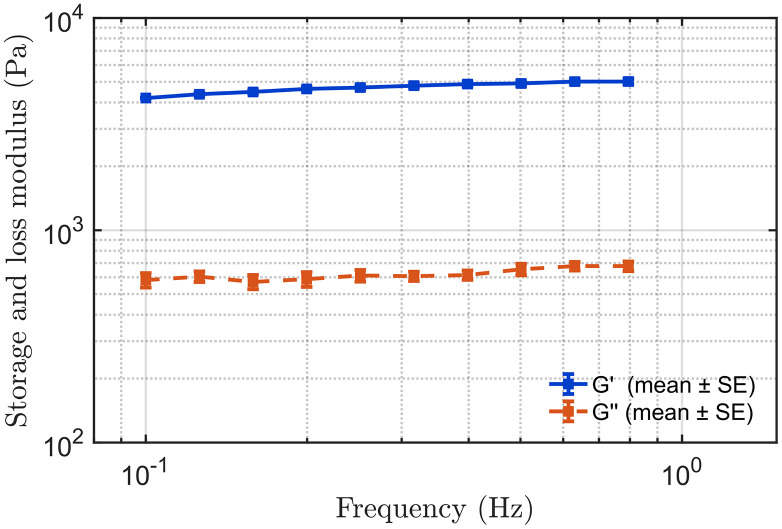
Storage modulus (G′) and loss modulus (G′′) of the hard tissue-mimicking phantom measured by rheometry. The square markers represent the mean values obtained from three independent measurements, while the horizontal error bars indicate the corresponding standard errors.

**Fig 13:**
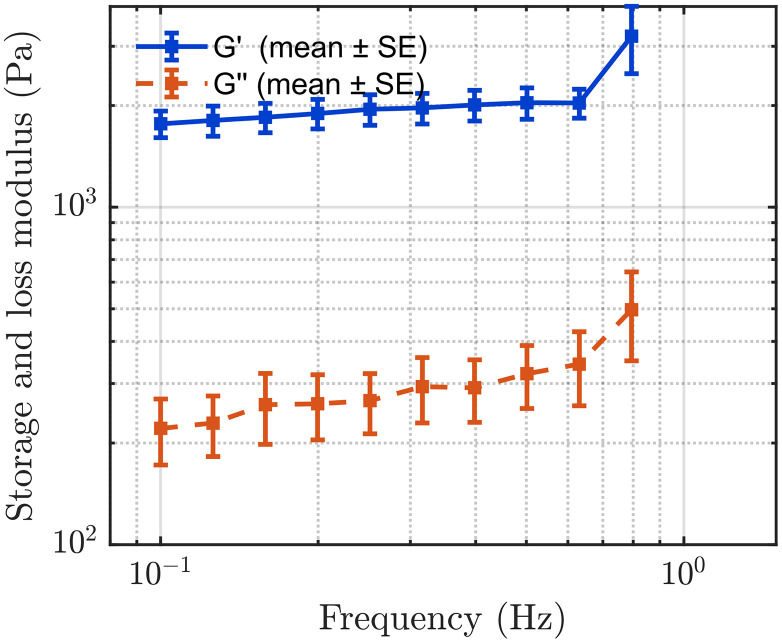
Storage modulus (G′) and loss modulus (G′′) of the soft tissue-mimicking phantom measured by rheometry. The square markers represent the mean values obtained from three independent measurements, while the horizontal error bars indicate the corresponding standard deviations.

Figs 12 and 13 present the mean storage modulus (G′) and loss modulus (G′′) of the complex shear modulus for the hard and soft tissue-mimicking phantoms, respectively, as measured by rheometry within their identified LVR. We tested each phantom in triplicate and calculated the mean values and the corresponding standard errors to assess measurement consistency and repeatability.

**Fig 14:**
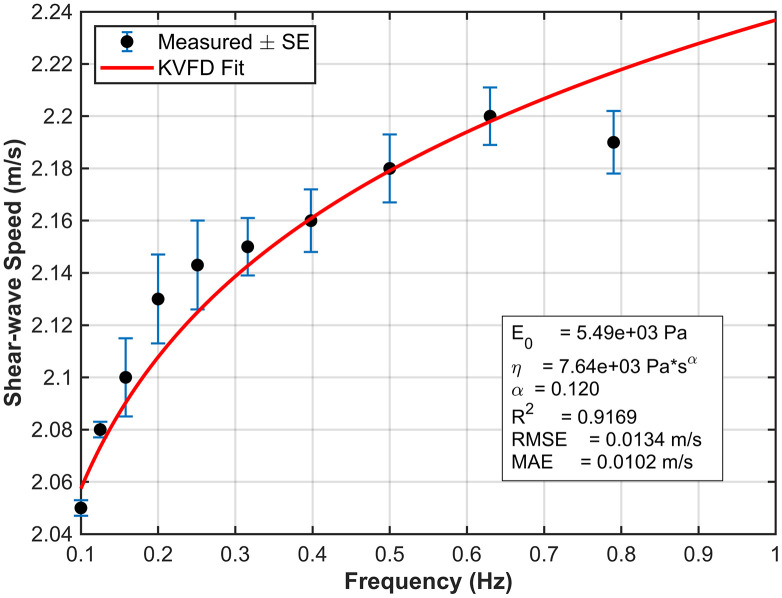
The KVFD model fits the dispersive data of the hard tissue-mimicking phantom across a frequency range of 0.1-1 Hz. Black data points represent the mean shear wave speed ± standard error from three rheometric measurements.

**Fig 15:**
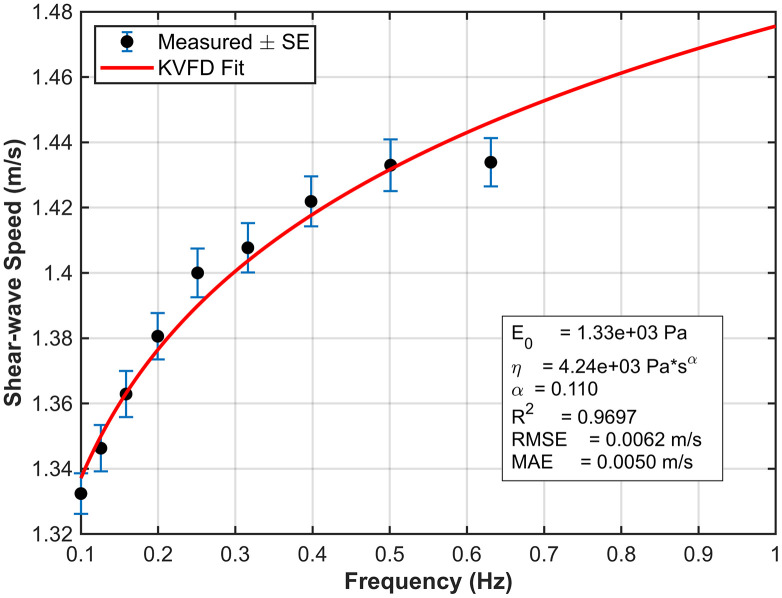
The KVFD model fits the dispersive data of the soft tissue-mimicking phantom across a frequency range of 0.1–1 Hz. Blue data points represent the mean shear wave speed ± standard error from three rheometric measurements.

To extract the numerical values of the parameters of interest, *E*_*o*_*, η,* and *α*, from the rheometer measurements, we fitted the dispersive data for each phantom using the KVFD model. The complex modulus ∣G*∣ was calculated from the G’ and G“ at each respective frequency. Shear wave velocity was then determined using equation 18. Figs 14 and 15 show the KVFD fits for the hard and soft tissue-mimicking phantoms.

**Fig 16:**
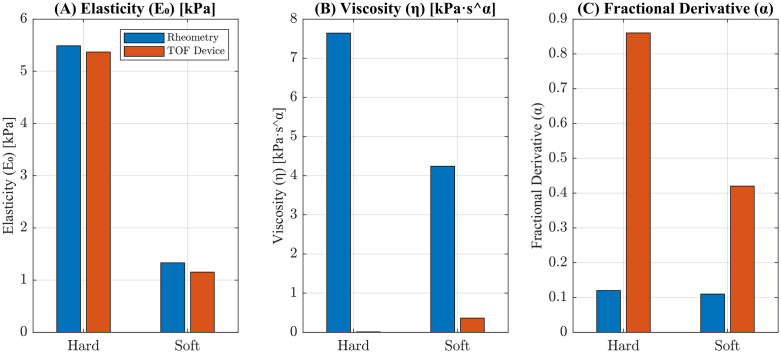
Comparison of mechanical property estimates between the standard rheometry technique and TOF-device for hard and soft tissue mimicking phantoms. (A) Elasticity (E_o_), (B) Viscosity (η), and (C) Fractional derivative (α) values shown for each phantom type.

Table 4 presents the numerical values obtained from each method. The bar plots (Fig 16) show close agreement between the TOF device and rheometry in estimating elasticity. However, we observed a noticeable difference in Viscosity and fractional derivative values, with greater deviations in the hard phantom.

**Table 4:** Absolute and relative error between the TOF-based device and rheometer measurements for elasticity (E_o_), viscosity (η), and the fractional derivative (α) in hard and soft tissue mimicking phantoms.

Phantom	Parameter	Rheometer	TOF Device	Abs error	Relative error
Hard	Elasticity *E*_*o*_ (kPa)	5.49	5.37	0.12	2.19%
Hard	Viscosity η (kPa.s^α^)	7.64	0.012	7.52	98.4%
Hard	Fractional derivative(α)	0.12	0.86	0.74	616.6%
Soft	Elasticity *E*_*o*_ (kPa)	1.33	1.152	0.18	13.38%
Soft	Viscosity η (kPa.s^α^)	4.24	0.0363	0.42	97.66%
Soft	Fractional derivative(α)	0.11	0.42	0.31	169.23%

Our findings indicate that the relaxed elastic modulus E₀ derived from the TOF measurements closely matches the rheometry estimates for both phantoms, as summarized in Table 4, despite the rheometer operating over a lower frequency range than the TOF device. In contrast, the viscosity η and fractional order α differed substantially between methods. Because η and α are strongly dependent on the measurement band, directly comparing these parameters across techniques with nonoverlapping frequency ranges is inappropriate. Instead, we report curve-level errors in the appropriate observable rather than directly equating parameters across disjoint bands. We therefore evaluated band-matched cross-projections of the KVFD fits in the velocity domain and performed an attenuation-related bias check under KVFD.

### 3.6 Band-matched cross projections

**Fig 17:**
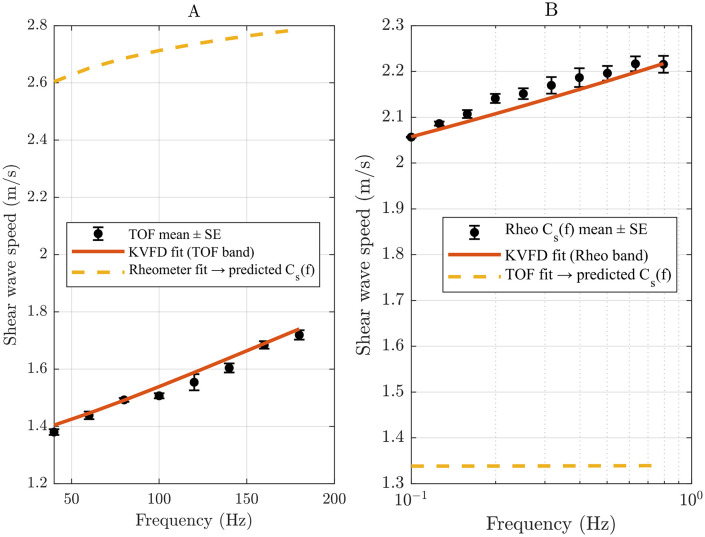
Cross-band velocity domain projection. (A) TOF band: Measured cs(f) with native TOF KVFD fit presented with red solid line and projected values from rheometer data presented with yellow dashed line. (B) Rheometer band: Measured cs(f) derived from G’ and G“ with native rheometer KVFD fit presented with red solid line and projected values from TOF data presented with yellow dashed line. The overlays illustrate the limited transferability of η and α across the frequency band.

**Fig 18:**
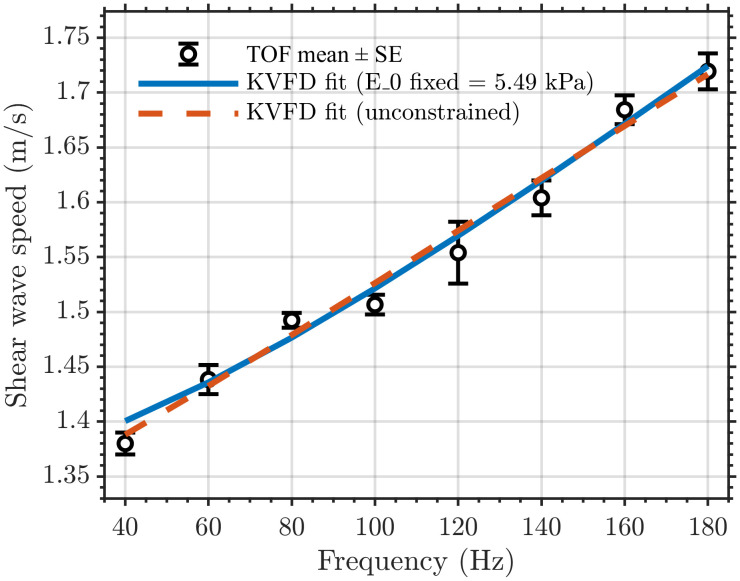
TOF dispersive data of the hard tissue-mimicking phantom fit with the KVFD model. The blue solid line represents a constrained refit with E_o _= 5.49 kPa, fixed to the rheometer value. The red dashed line represents an unconstrained TOF dispersive data fit.

Using the rheometer band fit with E_o_ = 5.49 kPa, η = 7.64 kPa·s^α, and α = 0.12, projection into 40–180 Hz over-predicted TOF shear wave speeds resulted in RMSE of 1.17 m/s, MAPE of 75.9%, and a mean bias of +1.17 m/s. Conversely, projecting the TOF band fit with E_o_ = 5.37 kPa, η = 0.012 kPa·s^α, and α = 0.86 into 0.1–1 Hz under predicted rheometer-derived velocities that resulted in RMSE of 0.816 m/s, MAPE of 37.8%, and mean bias of − 0.814 m/s. These outcomes show that η and α calibrated at low frequency do not transfer to the high-frequency propagation regime. The reverse is also true, even under a standard KVFD forward model, as shown in Fig 17. For a constrained TOF refit fixed to E_o_ = 5.49 kPa, re-optimizing η and α over 40–180 Hz yielded η = 0.00841 kPa·s^α and α = 0.909 with RMSE of 0.0139 m/s, reduced from 0.0237 m/s in the unconstrained fit. The dispersion error changed modestly, as shown in Fig 18. In contrast, η and α shifted appreciably, supporting the interpretation that differences in these parameters primarily reflect band sensitivity rather than suboptimal optimization.

### 3.7 Attenuation-related bias check under KVFD

Using the KVFD parameters for the hard phantom (Table 4), we computed *E′(f), E’‘(f)*, and the loss angle *δ(f)* = arctan *(E’‘/ E′)* over 40–180 Hz with a short path (i.e., sample length *D = 0.04 m*). δ increases smoothly from 13.70 at 40 Hz to 37.80 at 180 Hz, yielding a phase-velocity inflation factor **F(f) = 1/cos(*δ/*2)** that rises from 1.007 to 1.057, equivalent to 0.7–5.7% bias as shown in Table 5. The repeatability was high, as indicated by the three repeated measurements per frequency, with standard error (SE%) ranging from 0.5 to 1.8% and a coefficient of variation (CV%) of 0.8–3.1*%.* Thus, in the 40–140 Hz range, the damping-related inflation *(≤ 4.3%)* is within a few percent and comparable to the observed scatter, supporting a dispersion-only fit. At 160–80 Hz, the inflation remains modest (≈ 5–6%), which is still small relative to the mean speeds but exceeds SE, so any forward modeling that assumes lossless phase speed could optionally de-inflate by **cos (*δ/*2)** without materially altering conclusions. The magnitude of *δ* is consistent with prior soft-tissue data in the 20–200 Hz band and with polymer-gel measurements in the 1–100 Hz band, and the inflation relation follows directly from the viscoelastic wave speed expression [49–51]

**Table 5:** Summary of frequency-dependent measurements showing mean values (±SE), δ (^0^) correction factor (F), and the resulting percentage inflation across the 40 - 180 Hz bandwidth.

Frequency (Hz)	Mean ±SE	δ (^0^)	F	% inflation
40	1.380 ± 0.010	13.7	1.007	0.7
60	1.438 ± 0.013	18.6	1.013	1.3
80	1.492 ± 0.007	22.9	1.020	2.0
100	1.507 ± 0.009	26.6	1.028	2.8
120	1.5554 ± 0.028	29.9	1.035	3.5
140	1.604 ± 0.016	32.9	1.043	4.3
160	1.684 ± 0.013	35.5	1.050	5.0
180	1.719 ± 0.016	37.8	1.057	5.7

## 4. Discussion

Ormachea’s 30-year review highlights a persistent validation gap in elastography. DMA and rheometry remain the reference standards [16,52,13], but these are typically restricted to 0–100 Hz for in vitro and ex vivo testing [16]. Many elastography methods span the frequency range of 0–500 Hz

[53,54], resulting in a frequency mismatch that complicates validation. As a result, prior studies have relied on cross-validation despite fundamental differences in technique [27,55,56]. Our study addresses the validation gap by introducing and testing a novel short-path shear wave TOF device, together with a loss-aware KVFD model, for quantifying viscoelastic parameters in tissue-mimicking phantoms. We compared the standard rheometry technique with a novel TOF-based device for both hard and soft tissue-mimicking phantoms. Each modality was fitted in its native frequency band (TOF: 40–180 Hz; rheometer 0.1–1 Hz), and the fitted parameter sets are summarized in Table 4.

Elasticity measurements *(E*_*o*_*)* from the TOF device showed reasonable agreement with those from rheometry, supporting the transferability of *(E*_*o*_*)*, while viscous descriptors showed strong band sensitivity. Projecting rheometer parameters into the TOF band resulted in an RMSE of 1.17 m/s, a MAPE of 75.9%, and a positive bias of 1.17 m/s. Projecting TOF parameters into the rheometer band resulted in an RMSE of 0.816 m/s, a MAPE of 37.8%, and a negative bias of 0.814 m/s.

Anchoring rheometer *(E*_*o*_*)* preserved fit quality but shifted viscous parameter *(η, α)* substantially, indicating differences reflect true frequency regime sensitivity, not fitting artifacts. The parameters estimated from rheometer data at 0.1–1 Hz yielded higher viscosities and lower fractional orders than those from the TOF device at 40–180 Hz. This discrepancy reflects the intrinsic frequency-dependent nature of soft tissue viscoelasticity. Low-frequency measurements predominantly capture viscous effects; high-frequency techniques emphasize elastic and dispersive responses. As a result, the extracted parameters are specific to the experimental frequency range and cannot be compared across methods without accounting for this frequency dependence.

This finding aligns with the established consensus from prior cross-modal comparisons. Lin et al. compared SDUV (100–400 Hz) to rheometry (1–30 Hz) and reported consistent elasticity but significantly lower viscosity using the high-frequency method. Similarly, Callejas et al., using Torsional wave elastography (TWE, 300–1000 Hz) and a KVFD model, found that elastic terms were comparable across bands when fitting matched cervical data. Additionally, viscosity measured by high-frequency TWE better predicted the overall dispersive pattern than viscosity measured by low-frequency rheometry. Notably, KVFD α from TWE was close to 1, effectively collapsing to KV again, highlighting the band-sensitivity of *η* and *α*, which reinforces the notion that *η* and *α* are scale-dependent descriptors. Our hard and soft phantoms thus reinforce the consensus that *η* and *α* act as scale-dependent summaries of material behavior over a specific frequency window, whereas *E*_*o*_ is a more robust, band-stable property.

Measurements were taken at 26 °C with a controlled preload and identical lateral coupling, ensuring a far-field geometry **(D ≥ 2*λ)* and a boundary clearance of more than *λ*. The SE-weighted inversion method was employed, with repeatability typically meeting the criteria of *SE ≤ 2%* and *CV ≤ 3%.* A post-fit loss-consistency check revealed modest loss angles across the 40–180 Hz range and predicted phase-velocity inflation of only a few percent, comparable to measurement variability. Practically, these results support the use of TOF-derived elasticity for phantom standardization and calibration. However, viscous parameters should be interpreted within their measurement band or through fixed or joint analyses when cross-band comparability is required [34]

### 4.1 Sources of error and mitigation strategies

We identified and addressed several potential sources of error to ensure accurate and reliable measurements using the TOF-based system. One key factor was the alignment of the transmitting (Tx) and receiving (Rx) bimorph actuators. We used a mechanical rail and precision dial mechanism to position the Rx bimorph accurately relative to the sample, thereby minimizing misalignment and ensuring consistent placement. This approach reduces angular misalignment and geometric variability, which are known to introduce TOF errors [57].

Signal distortion and noise were essential challenges, particularly for detecting shear wave arrival times. We implemented a multi-stage signal processing pipeline to address this. The signal processing pipeline yielded a minor SNR improvement of 0.17dB. While modest in numerical terms, the processed signal demonstrated clearer temporal features and improved wavefront localization, both of which are essential for accurate TOF detection and viscoelastic parameter extraction. Consequently, the cumulative energy method could be applied more confidently and precisely.

We identified another potential error source in the measurement of propagation distance. To mitigate inaccuracies, we varied the distance between the Tx and Rx bimorphs in controlled increments of 0.5 mm, and repeated measurements at five discrete positions. As anticipated, the hard phantom exhibited a higher shear wave speed (Table 2) and lower TOF (Fig 9), consistent with its increased stiffness. Conversely, the soft phantom demonstrated a lower shear wave speed (Table 3) and higher TOF (Fig 10), reflecting its relatively lower stiffness. This observed relationship aligns with established biomechanical principles, which state that stiffer materials support the faster propagation of shear waves than softer ones [58,59]. The approach allowed for averaging across multiple trials, thereby reducing local bias and enhancing the overall robustness of the velocity measurements. The observed standard error values of shear wave speed further support these findings, ranging from 0.004 m/s to 0.006 m/s for the soft phantom and from 0.011 m/s to 0.021 m/s for the hard phantom across the studied frequency range of 40 Hz to 180 Hz. These narrow standard error margins highlight the consistency and reliability of the proposed TOF-based measurement technique.

Temperature also posed a potential source of error. The mechanical properties of soft tissues and biomaterials, such as PVA phantoms, are susceptible to temperature fluctuations [44]. During preliminary trials, we observed that inconsistent ambient conditions led to sample dehydration and variations in stiffness. We therefore matched the specimen setpoint at 26°C for both modalities. Before each acquisition, phantoms were allowed to equilibrate at 26°C. We carefully controlled the experimental environment by maintaining a room temperature of 26°C and limiting each session to one hour to avoid moisture and thermal drift during measurements. Under these matched conditions, any residual thermal bias is expected to be minor and cannot account for much larger cross-band projection differences, which we attribute to frequency-regime sensitivity of η and α. These precautions were essential to preserving the intrinsic viscoelastic behavior of the samples throughout the measurements.

### 4.2 Limitations

We acknowledge certain limitations despite the promising outcomes. We restricted the current system to testing phantoms with a minimum size of 40 mm. Below this threshold, signal overlap between the transmitter and receiver complicates TOF detection. The system’s minimum size requirement limits its use in clinical scenarios with small anatomical structures, such as lesions <10 mm. Additionally, we conducted the study using homogeneous artificial tissue, which, although suitable for validation, does not fully replicate the structural complexity of biological tissue, such as heterogeneity or anisotropy.

The study limited the dispersion frequency range to 40 Hz −180 Hz to ensure alignment with the standard rheometry range for validation purposes. We limited our test to a narrower bandwidth and did not utilize the system’s full operational range of 1000 Hz. Future efforts are needed to capitalize on this higher frequency potential.

## 5. Future work

We proposed future directions to address the limitations and expand the utility of the TOF system. First, increasing the transmission frequency would shorten the pulse length and extend the TOF window, thereby enabling higher resolution measurements, especially for smaller samples. Second, incorporating machine learning approaches such as deep learning models trained to recognize the onset of shear wave disturbance in noisy environments could improve TOF detection accuracy.

Additionally, future validation should include testing on heterogeneous, anisotropic, and biologically derived tissues to evaluate clinical applicability. This step is crucial for demonstrating the system’s effectiveness in real-world diagnostic environments.

As highlighted by Jotham et al., viscoelastic parameters, particularly viscosity and the fractional derivative order, are influenced by physiological conditions such as inflammation. Therefore, another promising direction would be to evaluate whether the TOF system, in conjunction with the KVFD model, can detect such pathological changes. If this capability is currently limited, algorithmic refinement will be necessary to enhance sensitivity to disease-related viscoelastic variations.

Future efforts should also explore real-time implementation of the signal processing pipeline to enhance the practical applicability of the system. Integrating embedded systems or FPGA-based platforms could enable efficient on-device computation, significantly improving the method’s translational potential for point-of-care diagnostics and clinical deployment.

### Conclusion

We present a short-path shear wave time-of-flight (TOF) system combined with a loss-aware Kelvin-Voigt derivative (KVFD) framework for rapid, benchtop quantification of viscoelastic parameters in ex vivo PVA phantoms, addressing a critical validation gap in elastography. The device produced reproducible elasticity estimates that agreed closely with rheometry (Eo within 2–13%), and it reliably discriminated between the hard and soft phantom types under controlled geometry, temperature (26°C), and preload.

At the same time, our cross-band analyses show that viscous descriptors (η) and the fractional order (α) are frequency regime dependent. Rheometer fits (0.1–1 Hz) returned large η and small α, whereas TOF fits (40–180 Hz) returned much smaller η and larger α. Band-matched projections and a constrained refit (anchoring *E*_*o*_) indicate that these differences reflect proper scale-sensitive rheology and measurement band effects rather than optimization failure. A post-fit loss consistency check confirmed modest loss angles and only small predicted phase-velocity inflation over the TOF band. Repeatability (SE, CV) and geometry checks support the method’s technical robustness.

Practically, TOF-derived elasticity is well-suited for phantom standardization and device calibration, but viscous parameters must be interpreted within their measurement band when cross-method comparison is required.

Importantly, the system’s proven robustness, non-destructive nature, and capacity for higher-frequency operation (up to 1000 Hz) provide a solid foundation for future work. We strongly advocate for further studies to extend this approach to real, heterogeneous biological tissues. Such research is essential to validate the method’s performance in clinically relevant scenarios, investigate its sensitivity to pathological states, and fully realize its potential as a high-resolution diagnostic tool for characterizing soft tissue.

## Supporting information

S1 File Dispersive data from rheometer for both phantoms. (XLSX)

S2 File Reported MATLAB data code. (ZIP)
