# Supplementary material for: Establishing an Elastography calibration standard: Validation of a shear wave TOF device for measuring Elasticity and Viscosity in tissue-mimicking phantoms using rheometry
Source: PLoS One. 2025 Nov 13;20(11):e0335645. doi: 10.1371/journal.pone.0335645 (PMC12614516; doi:10.1371/journal.pone.0335645)
Supplement: S2 File — (ZIP) [file pone.0335645.s002.zip › TOF_Data_fitting_with_tableofresults_Hard_Tissue.docx]

clear; clc;

%% === DATA ===

frequency = [40, 60, 80, 100, 120, 140, 160, 180];

Time1 = [0.02983, 0.02871, 0.02743, 0.02718, 0.02680, 0.02572, 0.02443, 0.02394];

Time2 = [0.02954, 0.02844, 0.02742, 0.02729, 0.02665, 0.02562, 0.02432, 0.02396];

Time3 = [0.02989, 0.02851, 0.02769, 0.02729, 0.02584, 0.02546, 0.02437, 0.02375];

Time4 = [0.02985, 0.02815, 0.02766, 0.02711, 0.02609, 0.02528, 0.02450, 0.02345];

Time5 = [0.02968, 0.02852, 0.02759, 0.02721, 0.02676, 0.02538, 0.02446, 0.02360];

Distance1 = 0.04056 * ones(1, length(frequency));

Distance2 = 0.04106 * ones(1, length(frequency));

Distance3 = 0.04156 * ones(1, length(frequency));

Distance4 = 0.04206 * ones(1, length(frequency));

Distance5 = 0.04256 * ones(1, length(frequency));

% Calculate velocities

Velocity1 = Distance1 ./ Time1;

Velocity2 = Distance2 ./ Time2;

Velocity3 = Distance3 ./ Time3;

Velocity4 = Distance4 ./ Time4;

Velocity5 = Distance5 ./ Time5;

% Stack for statistics

velocities = [Velocity1; Velocity2; Velocity3; Velocity4; Velocity5];

MeanVelocity = mean(velocities, 1);

std_Velocity = std(velocities, 0, 1);

N = size(velocities,1);

SE_Velocity = std_Velocity / sqrt(N);

%% === TASK 1: Display Table in Console ===

disp(' ');

disp('--------------------------------------------------------------------------');

disp(' Freq V1 V2 V3 Mean +/- SE (m/s)');

disp('--------------------------------------------------------------------------');

for i = 1:length(frequency)

fprintf('%8d %6.3f %6.3f %6.3f %7.3f +/- %.3f\n', ...

frequency(i), Velocity1(i), Velocity2(i), Velocity3(i), ...

MeanVelocity(i), SE_Velocity(i));

end

disp('--------------------------------------------------------------------------');

disp(' ');

% Prepare table for export

tableOut = table(frequency(:), Velocity1(:), Velocity2(:), Velocity3(:), ...

MeanVelocity(:), SE_Velocity(:), ...

'VariableNames', {'Frequency_Hz','Velocity1_m_per_s','Velocity2_m_per_s','Velocity3_m_per_s','MeanVelocity_m_per_s','SE_m_per_s'});

% For console (optional: formatted "mean +/- SE" string column)

formatted = strings(numel(frequency),1);

for i=1:numel(frequency)

formatted(i) = sprintf('%.3f +/- %.3f', MeanVelocity(i), SE_Velocity(i));

end

tableOut.Mean_SE_str = formatted;

%% === TASK 2: Publication-Quality Plot + KVFD Fit (COMPACT FORM) ===

rho = 1000;

f = frequency(:); % column vector for safety

y = MeanVelocity(:);

sem = SE_Velocity(:);

% Guard against zero SEM in weighting

if any(sem == 0)

nz = sem(sem > 0);

if isempty(nz)

sem(:) = 1e-2; % conservative fallback

else

sem(sem == 0) = max(1e-3, min(nz));

end

end

sqrtW = 1 ./ sem; % sqrt of 1/SEM^2

% --- Compact KVFD model from your screenshot ---

% Q(p,f) = E0^2 + 2*E0*eta*cos(pi*alpha/2)*(2*pi*f)^alpha + [eta*(2*pi*f)^alpha]^2

Q = @(p,ff) ( p(1)^2 ...

+ 2*p(1)*p(2).*cos(pi*p(3)/2).*(2*pi*ff).^p(3) ...

+ (p(2).^2).*(2*pi*ff).^(2*p(3)) );

% Cs(f) = sqrt( 2*Q / ( 3*rho * ( sqrt(Q) + E0 + eta*(2*pi*f)^alpha*cos(pi*alpha/2) ) ) )

curve_fit_func = @(p,ff) sqrt( 2.*Q(p,ff) ./ ( 3*rho .* ( sqrt(Q(p,ff)) ...

+ p(1) + p(2).*cos(pi*p(3)/2).*(2*pi*ff).^p(3) ) ) );

% Weighted model for lsqcurvefit: minimize sum(((yhat - y)./SEM).^2)

model_w = @(p,ff) curve_fit_func(p,ff(:)) .* sqrtW;

y_w = y .* sqrtW;

params_initial = [4.17234, 60.543347, 0.5]; % [E0 (Pa), eta (Pa*s^alpha), alpha]

lb = [0, 0, 0.4]; ub = [Inf, Inf, 0.99];

options = optimoptions('lsqcurvefit', 'MaxFunctionEvaluations', 2000, ...

'MaxIterations', 2000, 'Display', 'off');

[fit_params, resnorm, residual_w, exitflag, output, lambda, Jw] = ...

lsqcurvefit(model_w, params_initial, f, y_w, lb, ub, options);

% Predictions and metrics (unweighted)

v_pred = curve_fit_func(fit_params, f);

SSE = sum((y - v_pred).^2);

Rsq = 1 - SSE/sum((y - mean(y)).^2);

RMSE = sqrt(SSE/numel(f));

%% === 95% Confidence Intervals (robust; handles sparse J) ===

dof = max(1, numel(y) - numel(fit_params));

res_std = (y - v_pred) ./ sem; % standardized residuals

sigma2w = sum(res_std.^2) / dof; % == resnorm/dof

Jfull = full(Jw); % ensure dense

A = Jfull.'*Jfull;

[RR,pd] = chol(A);

if pd == 0

iA = RR \ (RR' \ eye(size(A)));

else

iA = pinv(A); % fallback if near-singular

end

Cov_p = sigma2w * iA;

SE_p = sqrt(diag(Cov_p));

z95 = 1.96;

CI_E0 = [fit_params(1)-z95*SE_p(1), fit_params(1)+z95*SE_p(1)];

CI_eta = [fit_params(2)-z95*SE_p(2), fit_params(2)+z95*SE_p(2)];

CI_alp = [fit_params(3)-z95*SE_p(3), fit_params(3)+z95*SE_p(3)];

%% === Plot: data with error bars and fitted curve ===

fig = figure('Units','inches','Position',[2 2 7 5],'Color','w'); hold on;

errorbar(frequency, MeanVelocity, SE_Velocity, 'o', ...

'MarkerFaceColor','b','Color',[0 0.45 0.74],'LineWidth', 2);

f_fine = linspace(min(f), max(f), 400);

plot(f_fine, curve_fit_func(fit_params, f_fine), 'r-', 'LineWidth', 2.8);

set(gca,'FontName','Arial','FontSize',13,'LineWidth',1.3);

xlabel('Frequency (Hz)', 'FontWeight','bold', 'FontSize', 15);

ylabel('Velocity (m/s)', 'FontWeight','bold', 'FontSize', 15);

legend('Mean +/- SE', 'KVFD Fit', 'Location', 'northwest', 'FontSize',13);

grid on; box on;

title('KVFD Model Fit to Velocity vs. Frequency','FontSize',16,'FontWeight','bold');

% Annotate extracted params and performance (ASCII only)

annotationStr = sprintf([ ...

'E_0 = %.2e Pa\n' ...

'\\eta = %.2e Pa*s^{\\alpha}\n' ...

'\\alpha = %.2f\n' ...

'R^2 = %.3f, RMSE = %.3f m/s'], ...

fit_params(1), fit_params(2), fit_params(3), Rsq, RMSE);

x_annot = min(f) + 0.60*(max(f)-min(f));

y_annot = min(MeanVelocity) + 0.83*(max(MeanVelocity)-min(MeanVelocity));

text(x_annot, y_annot, annotationStr, 'FontSize',12, ...

'BackgroundColor','w', 'EdgeColor','k', 'Margin',6, 'Interpreter','tex');

%% === Residuals plot (standardized by SEM) ===

fig2 = figure('Units','inches','Position',[10 2 7 3.2],'Color','w'); hold on;

plot(frequency, res_std, 'ks-','LineWidth',1.2,'MarkerFaceColor','k');

% reference lines (no yline to avoid version issues)

plot([min(frequency) max(frequency)], [0 0], 'k-');

plot([min(frequency) max(frequency)], [2 2], 'k--');

plot([min(frequency) max(frequency)], [-2 -2], 'k--');

xlabel('Frequency (Hz)','FontSize',12,'FontWeight','bold');

ylabel('Standardized Residual','FontSize',12,'FontWeight','bold');

set(gca,'FontName','Arial','FontSize',11,'LineWidth',1.1); grid on; box on;

%% === Export plots ===

saveas(fig, 'Velocity_Fit_PubQuality.fig');

exportgraphics(fig, 'Velocity_Fit_PubQuality.png', 'Resolution', 600, 'BackgroundColor', 'white');

exportgraphics(fig, 'Velocity_Fit_PubQuality.eps', 'ContentType', 'vector');

exportgraphics(fig2, 'Velocity_Fit_Residuals.png', 'Resolution', 600);

%% === TASK 3: Export Data Tables ===

writetable(tableOut, 'VelocityTable.csv');

writetable(tableOut, 'VelocityTable.xlsx');

%% === TASK 4: Export Fit Parameters and Performance (with CIs) ===

params_table = table( ...

{'E0_Pa'; 'Eta_Pa_s_alpha'; 'Alpha'; 'R2'; 'RMSE_m_per_s'; ...

'E0_CI_low'; 'E0_CI_high'; 'Eta_CI_low'; 'Eta_CI_high'; 'Alpha_CI_low'; 'Alpha_CI_high'}, ...

[fit_params(1); fit_params(2); fit_params(3); Rsq; RMSE; ...

CI_E0(1); CI_E0(2); CI_eta(1); CI_eta(2); CI_alp(1); CI_alp(2)], ...

'VariableNames', {'Parameter','Value'});

writetable(params_table, 'FitParameters_and_Performance.csv');

writetable(params_table, 'FitParameters_and_Performance.xlsx');

disp(' ');

disp('All plots and tables have been exported for download.');

disp(' ');
