# Supplementary material for: Establishing an Elastography calibration standard: Validation of a shear wave TOF device for measuring Elasticity and Viscosity in tissue-mimicking phantoms using rheometry
Source: PLoS One. 2025 Nov 13;20(11):e0335645. doi: 10.1371/journal.pone.0335645 (PMC12614516; doi:10.1371/journal.pone.0335645)
Supplement: S2 File — (ZIP) [file pone.0335645.s002.zip › TOF_Data_fitting_with_tableofresults_Soft_Tissue.docx]

%% Full KVFD Fit Script with All Tasks and Bottom-Right Annotation

clear; clc; close all

%% === DATA INPUT ===

frequency = [40, 60, 80, 100,120,140,160];

Time1 = [0.07128,0.06999,0.06860,0.06687,0.06648,0.06558,0.06549];

Time2 = [0.07154,0.06973,0.06829,0.06743,0.06685,0.06634,0.06556];

Time3 = [0.07134,0.06967,0.06820,0.06768,0.06679,0.06646,0.06552];

Time4 = [0.07155,0.06954,0.06848,0.06768,0.06647,0.06640,0.06546];

Time5 = [0.07163,0.07000,0.06842,0.06693,0.06674,0.06637,0.06547];

D1 = 0.04891; D2 = 0.04941; D3 = 0.04991; D4 = 0.05041; D5 = 0.05091;

Distance1 = D1*ones(1,numel(frequency));

Distance2 = D2*ones(1,numel(frequency));

Distance3 = D3*ones(1,numel(frequency));

Distance4 = D4*ones(1,numel(frequency));

Distance5 = D5*ones(1,numel(frequency));

%% === 1. Compute Velocities & Stats ===

V1 = Distance1./Time1; V2 = Distance2./Time2; V3 = Distance3./Time3;

V4 = Distance4./Time4; V5 = Distance5./Time5;

vel = [V1; V2; V3; V4; V5];

MeanV = mean(vel,1);

StdV = std(vel,0,1);

N = size(vel,1);

SE_V = StdV/sqrt(N);

%% === TASK 1: Display Table in Console ===

fprintf('\n=== Velocity Statistics ===\n');

fprintf('Freq(Hz) V1(m/s) V2(m/s) V3(m/s) V4(m/s) V5(m/s) Mean+/-SE (m/s)\n');

for i=1:numel(frequency)

fprintf('%8d %7.4f %7.4f %7.4f %7.4f %7.4f %7.4f +/- %.4f\n', ...

frequency(i), V1(i),V2(i),V3(i),V4(i),V5(i), MeanV(i), SE_V(i));

end

tableOut = table(frequency(:),V1(:),V2(:),V3(:),V4(:),V5(:),MeanV(:),SE_V(:), ...

'VariableNames',{'Frequency_Hz','V1','V2','V3','V4','V5','MeanVelocity','SE_mps'});

%% === 2. KVFD MODEL FIT (compact form) ===

rho = 1000;

f = frequency(:); % column vector

y = MeanV(:);

sem = SE_V(:);

% Guard against zero SEM for weighting

if any(sem==0)

nz = sem(sem>0);

sem(sem==0) = max(1e-3, (isempty(nz)*1e-2) + (~isempty(nz))*min(nz));

end

sqrtW = 1 ./ sem; % sqrt of 1/SEM^2

% Q(p,f) = E0^2 + 2*E0*eta*cos(pi*alpha/2)*(2*pi*f)^alpha + [eta*(2*pi*f)^alpha]^2

Q = @(p,ff) ( p(1)^2 ...

+ 2*p(1)*p(2).*cos(pi*p(3)/2).*(2*pi*ff).^p(3) ...

+ (p(2).^2).*(2*pi*ff).^(2*p(3)) );

% Cs(f) = sqrt( 2*Q / ( 3*rho * ( sqrt(Q) + E0 + eta*(2*pi*f)^alpha*cos(pi*alpha/2) ) ) )

kvfd = @(p,ff) sqrt( 2.*Q(p,ff) ./ ( 3*rho .* ( sqrt(Q(p,ff)) ...

+ p(1) + p(2).*cos(pi*p(3)/2).*(2*pi*ff).^p(3) ) ) );

% Weighted least squares via lsqcurvefit trick

model_w = @(p,ff) kvfd(p,ff(:)) .* sqrtW;

y_w = y .* sqrtW;

p0 = [1.86296, 0.158966, 0.5]; % [E0 (Pa), eta (Pa*s^alpha), alpha]

lb = [0,0,0.42]; ub = [Inf,Inf,0.99];

opts = optimoptions('lsqcurvefit','Display','off','MaxIterations',2000);

try, opts = optimoptions(opts,'MaxFunctionEvaluations',1e5); catch, end

[pf,resnorm,~,exitflag,output,~,Jw] = ...

lsqcurvefit(model_w,p0,f,y_w,lb,ub,opts);

% Predictions and metrics (unweighted)

vfit = kvfd(pf,f);

res = y - vfit;

SSE = sum(res.^2);

R2 = 1 - SSE/sum((y-mean(y)).^2);

RMSE = sqrt(mean(res.^2));

MAE = mean(abs(res));

res_std = res ./ sem;

%% === 95% CIs (robust; handles sparse J) ===

dof = max(1, numel(y) - numel(pf));

sigma2w = sum(res_std.^2) / dof; % == resnorm/dof

Jfull = full(Jw); % ensure dense

A = Jfull.'*Jfull;

[RR,pd] = chol(A);

if pd==0

iA = RR \ (RR' \ eye(size(A)));

else

iA = pinv(A);

end

Cov_p = sigma2w * iA;

SE_p = sqrt(diag(Cov_p));

z95 = 1.96;

CI_E0 = [pf(1)-z95*SE_p(1), pf(1)+z95*SE_p(1)];

CI_eta = [pf(2)-z95*SE_p(2), pf(2)+z95*SE_p(2)];

CI_alp = [pf(3)-z95*SE_p(3), pf(3)+z95*SE_p(3)];

%% === TASK 2: Publication-Quality Plot (no blue connecting line) ===

h = figure('Units','inches','Position',[1 1 7 5],'Color','w');

hold on; grid on; box on

% Data: markers with caps only

errorbar(f, y, sem, 'o', ...

'MarkerFaceColor',[0 0.45 0.74], ...

'MarkerEdgeColor',[0 0.45 0.74], ...

'LineStyle','none', 'CapSize',10, ...

'DisplayName','Mean \pm SE');

% Fit line

f_fine = linspace(min(f),max(f),400);

plot(f_fine, kvfd(pf,f_fine), 'r-','LineWidth',2.5,'DisplayName','KVFD Fit');

set(gca,'FontName','Arial','FontSize',13,'LineWidth',1.2);

xlabel('Frequency (Hz)','FontSize',15,'FontWeight','bold');

ylabel('Velocity (m/s)','FontSize',15,'FontWeight','bold');

title('KVFD Model Fit to Velocity vs. Frequency','FontSize',16,'FontWeight','bold');

legend('Location','northwest','FontSize',12,'Box','off');

% Annotation bottom-right (TeX-safe)

ann = sprintf([ ...

'E_0 = %.3e Pa\n' ...

'\\eta = %.3e Pa\\cdot s^{\\alpha}\n' ...

'\\alpha = %.3f\n' ...

'R^2 = %.3f\n' ...

'RMSE = %.3e m/s\n' ...

'MAE = %.3e m/s'], ...

pf(1), pf(2), pf(3), R2, RMSE, MAE);

annotation('textbox',[0.60 0.12 0.36 0.42], ...

'String',ann, 'FitBoxToText','on', 'BackgroundColor','w', ...

'Interpreter','tex', 'FontSize',10.5, 'EdgeColor','k');

%% === Residuals plot (standardized by SEM) ===

h2 = figure('Units','inches','Position',[8.2 1 7 3.2],'Color','w'); hold on;

plot(frequency, res_std, 'ks-','LineWidth',1.2,'MarkerFaceColor','k');

plot([min(frequency) max(frequency)], [0 0], 'k-');

plot([min(frequency) max(frequency)], [2 2], 'k--');

plot([min(frequency) max(frequency)], [-2 -2], 'k--');

xlabel('Frequency (Hz)','FontSize',12,'FontWeight','bold');

ylabel('Standardized Residual','FontSize',12,'FontWeight','bold');

set(gca,'FontName','Arial','FontSize',11,'LineWidth',1.1); grid on; box on;

%% === TASK 3: Export Data Tables ===

writetable(tableOut,'VelocityTable.csv');

writetable(tableOut,'VelocityTable.xlsx');

%% === TASK 4: Export Fit Parameters & Performance ===

params = {'E0_Pa';'Eta_Pa_s_alpha';'Alpha';'R2';'RMSE_m_s';'MAE_m_s'; ...

'E0_CI_low';'E0_CI_high';'Eta_CI_low';'Eta_CI_high';'Alpha_CI_low';'Alpha_CI_high'};

values = [pf(1);pf(2);pf(3);R2;RMSE;MAE; CI_E0(1);CI_E0(2); CI_eta(1);CI_eta(2); CI_alp(1);CI_alp(2)];

params_table = table(params,values,'VariableNames',{'Parameter','Value'});

writetable(params_table,'FitParameters_and_Performance.csv');

writetable(params_table,'FitParameters_and_Performance.xlsx');

%% === EXPORT FIGURES ===

saveas(h,'Velocity_KVFD_Fit.fig');

exportgraphics(h,'Velocity_KVFD_Fit.png','Resolution',600);

exportgraphics(h,'Velocity_KVFD_Fit.eps','ContentType','vector');

exportgraphics(h2,'Velocity_KVFD_Residuals.png','Resolution',600);

disp(' '); disp('All tables and figures have been generated and exported.');
