# Supplementary material for: Establishing an Elastography calibration standard: Validation of a shear wave TOF device for measuring Elasticity and Viscosity in tissue-mimicking phantoms using rheometry
Source: PLoS One. 2025 Nov 13;20(11):e0335645. doi: 10.1371/journal.pone.0335645 (PMC12614516; doi:10.1371/journal.pone.0335645)
Supplement: S2 File — (ZIP) [file pone.0335645.s002.zip › Comparison_plot1.docx]

% Data

phantoms = {'Hard', 'Soft'};

%params = {'Elasticity (E₀) [kPa]', 'Viscosity (η) [kPa·s^{\alpha}]', 'Fractional Derivative (\alpha)'};

params = {'Elasticity (E₀) [kPa]', 'Viscosity (η) [kPa·s^α]', 'Fractional Derivative (α)'};

% Rheometry and TOF means

rheo_data = [5.49, 7.64, 0.12;

1.33, 4.24, 0.11];

tof_data = [5.37, 0.012, 0.86;

1.152, 0.363, 0.42];

% Combine data for plotting

methods = {'Rheometry', 'TOF Device'};

colors = [0 0.45 0.74; 0.85 0.33 0.1]; % Blue and red

figure('Units','inches','Position',[1 1 10 4]) % publication-friendly size

for i = 1:3

subplot(1,3,i)

% Data matrix: rows = phantom types, cols = methods

data = [rheo_data(:,i), tof_data(:,i)];

% Plot grouped bar chart

b = bar(data, 'grouped');

hold on

for k = 1:2

b(k).FaceColor = colors(k,:);

end

set(gca, 'XTickLabel', phantoms, 'FontSize', 11)

ylabel(params{i}, 'FontSize', 11)

title(['(', char('A'+i-1), ') ' params{i}], 'FontSize', 12)

if i == 1

legend(methods, 'Location', 'northeast', 'FontSize', 8)

end

grid on

end

% Save figure

print(gcf, 'TOF_vs_Rheometry_Comparison', '-dtiff', '-r600'); % High-resolution .tif

print(gcf, 'TOF_vs_Rheometry_Comparison', '-depsc'); % Vector .eps
