# Supplementary material for: Establishing an Elastography calibration standard: Validation of a shear wave TOF device for measuring Elasticity and Viscosity in tissue-mimicking phantoms using rheometry
Source: PLoS One. 2025 Nov 13;20(11):e0335645. doi: 10.1371/journal.pone.0335645 (PMC12614516; doi:10.1371/journal.pone.0335645)
Supplement: S2 File — (ZIP) [file pone.0335645.s002.zip › Cross_projection.docx]

%% two_panel_velocity_domain.m

% Panel A: ToF c_p(f): ToF fit + Rheometer->ToF projection (loss-aware)

% Panel B: Rheometer c_p(f): Rheometer fit + ToF->Rheometer projection (loss-aware)

% Also prints band-level RMSE/MAPE/bias summary.

clear; clc; close all;

rho = 1000; % kg/m^3

% ------- KVFD parameters (E-domain) -------

% ToF-band fit (40–180 Hz)

E0_tof = 5.37; eta_tof = 0.012; alpha_tof = 0.86;

% Rheometer-band fit (0.1–0.794 Hz)

E0_rheo = 5.49; eta_rheo = 7.64; alpha_rheo = 0.12;

% ------- ToF data (3 repeats) -------

f_tof = [40 60 80 100 120 140 160 180]';

cp_reps = [...

1.360 1.390 1.390;

1.413 1.444 1.458;

1.479 1.497 1.501;

1.492 1.505 1.523;

1.513 1.541 1.608;

1.577 1.603 1.632;

1.660 1.688 1.705;

1.694 1.714 1.750];

cp_tof_mean = mean(cp_reps,2);

cp_tof_SE = std(cp_reps,0,2)/sqrt(size(cp_reps,2));

% ------- Rheometer G', G'' (MPa), 3 sweeps -------

f_rheo = [0.1 0.125893 0.158489 0.199526 0.251189 0.316228 0.398107 0.501187 0.630957 0.794328]';

G1p = [4.18E-03 4.28E-03 4.36E-03 4.50E-03 4.53E-03 4.58E-03 4.64E-03 4.70E-03 4.78E-03 4.77E-03]';

G1pp = [5.30E-04 5.56E-04 4.99E-04 4.98E-04 5.43E-04 5.58E-04 5.54E-04 5.87E-04 6.24E-04 6.07E-04]';

G2p = [4.17E-03 4.33E-03 4.46E-03 4.61E-03 4.67E-03 4.81E-03 4.90E-03 4.90E-03 4.99E-03 5.00E-03]';

G2pp = [5.43E-04 5.86E-04 5.62E-04 6.06E-04 6.13E-04 6.00E-04 6.32E-04 6.46E-04 6.74E-04 7.00E-04]';

G3p = [0.0041849 0.0042777 0.00436008 0.00450072 0.00452834 0.00457605 0.00464437 0.00469528 0.00478207 0.00476954]';

G3pp = [0.000529981 0.000555518 0.0004989 0.000498435 0.000542852 0.000557523 0.000553707 0.000587187 0.00062356 0.00060691]';

% Convert MPa->Pa for loss-aware velocity from G', G''

G1p_Pa = G1p * 1e6; G1pp_Pa = G1pp * 1e6;

G2p_Pa = G2p * 1e6; G2pp_Pa = G2pp * 1e6;

G3p_Pa = G3p * 1e6; G3pp_Pa = G3pp * 1e6;

% Rheometer c_p per sweep (loss-aware), then mean ± SE

cp_rheo_1 = map_cp_from_G(G1p_Pa, G1pp_Pa, rho);

cp_rheo_2 = map_cp_from_G(G2p_Pa, G2pp_Pa, rho);

cp_rheo_3 = map_cp_from_G(G3p_Pa, G3pp_Pa, rho);

cp_rheo_mat = [cp_rheo_1, cp_rheo_2, cp_rheo_3];

cp_rheo_mean = mean(cp_rheo_mat,2);

cp_rheo_SE = std(cp_rheo_mat,0,2)/sqrt(3);

% ------- Predictions for plots (loss-aware) -------

% ToF panel

[Ep_tof, Epp_tof] = kvfd_E(f_tof, E0_tof, eta_tof, alpha_tof);

[Ep_r2t, Epp_r2t] = kvfd_E(f_tof, E0_rheo, eta_rheo, alpha_rheo);

cp_fit_ToF = map_cp_from_E(Ep_tof, Epp_tof, rho); % ToF native fit

cp_pred_rheo2tof = map_cp_from_E(Ep_r2t, Epp_r2t, rho); % Rheo->ToF projection

% Rheometer panel

[Ep_rheo, Epp_rheo] = kvfd_E(f_rheo, E0_rheo, eta_rheo, alpha_rheo); % Rheo native

[Ep_t2r, Epp_t2r] = kvfd_E(f_rheo, E0_tof, eta_tof, alpha_tof); % ToF->Rheo projection

cp_fit_Rheo = map_cp_from_E(Ep_rheo, Epp_rheo, rho);

cp_pred_tof2rheo = map_cp_from_E(Ep_t2r, Epp_t2r, rho);

% ------- Summary metrics (velocity domain) -------

% Rheo->ToF

rmse_r2t = rmse(cp_pred_rheo2tof, cp_tof_mean);

mape_r2t = 100*mean(abs(cp_pred_rheo2tof - cp_tof_mean)./cp_tof_mean);

bias_r2t = mean(cp_pred_rheo2tof - cp_tof_mean);

% ToF->Rheo

rmse_t2r = rmse(cp_pred_tof2rheo, cp_rheo_mean);

mape_t2r = 100*mean(abs(cp_pred_tof2rheo - cp_rheo_mean)./cp_rheo_mean);

bias_t2r = mean(cp_pred_tof2rheo - cp_rheo_mean);

fprintf('Rheometer -> ToF: RMSE = %.3f m/s | MAPE = %.1f %% | Mean bias = %+0.3f m/s\n',...

rmse_r2t, mape_r2t, bias_r2t);

fprintf('ToF -> Rheometer: RMSE = %.3f m/s | MAPE = %.1f %% | Mean bias = %+0.3f m/s\n',...

rmse_t2r, mape_t2r, bias_t2r);

% ------- Two-panel figure -------

t = tiledlayout(1,2,'Padding','compact','TileSpacing','compact');

% (A) ToF: measured vs ToF fit and Rheo->ToF projection

nexttile; hold on; box on; grid on;

errorbar(f_tof, cp_tof_mean, cp_tof_SE, 'k.','MarkerSize',14,'LineWidth',1.0,'DisplayName','TOF mean ± SE');

plot(f_tof, cp_fit_ToF, '-','LineWidth',2.0,'DisplayName','KVFD fit (TOF band)');

plot(f_tof, cp_pred_rheo2tof, '--','LineWidth',2.0,'DisplayName','Rheometer fit → predicted C_s(f)');

xlabel('Frequency (Hz)'); ylabel('Shear wave speed (m/s)');

title('A');

legend('Location','east'); set(gca,'FontName','Times New Roman','FontSize',9);

h_legend = legend;

set(h_legend, 'Interpreter', 'tex');

% (B) Rheometer: measured (mean±SE) vs Rheo fit and ToF->Rheo projection

nexttile; hold on; box on; grid on;

errorbar(f_rheo, cp_rheo_mean, cp_rheo_SE, 'k.','MarkerSize',14,'LineWidth',1.0,'DisplayName','Rheo C_s(f) mean ± SE');

plot(f_rheo, cp_fit_Rheo, '-','LineWidth',2.0,'DisplayName','KVFD fit (Rheo band)');

plot(f_rheo, cp_pred_tof2rheo,'--','LineWidth',2.0,'DisplayName','TOF fit → predicted C_s(f)');

set(gca,'XScale','log');

xlabel('Frequency (Hz)'); ylabel('Shear wave speed (m/s)');

title('B');

legend('Location','east'); set(gca,'FontName','Times New Roman','FontSize',10);

h_legend = legend;

set(h_legend, 'Interpreter', 'tex');

%title(t, 'Cross-band velocity-domain projections under KVFD','FontWeight','bold');

% Save

outdir = fullfile(pwd,'figures'); if ~isfolder(outdir), mkdir(outdir); end

base = fullfile(outdir,'two_panel_velocity_domain');

set(gcf,'PaperPositionMode','auto');

if exist('exportgraphics','file')==2

exportgraphics(gcf,[base '.pdf'],'ContentType','vector','BackgroundColor','white');

exportgraphics(gcf,[base '.png'],'Resolution',600,'BackgroundColor','white');

else

print(gcf,[base '.pdf'],'-dpdf','-painters');

print(gcf,[base '.png'],'-dpng','-r600');

end

disp('Saved: ./figures/two_panel_velocity_domain.[pdf|png]');

%% ----------------- Helpers -----------------

function [Ep,Epp] = kvfd_E(f_Hz, E0_kPa, eta_kPa_s_a, alpha)

w = 2*pi*f_Hz(:);

pow = w.^alpha;

c = cos(pi*alpha/2); s = sin(pi*alpha/2);

Ep = E0_kPa + eta_kPa_s_a .* pow .* c; % kPa

Epp = eta_kPa_s_a .* pow .* s; % kPa

end

function cp = map_cp_from_E(Ep_kPa, Epp_kPa, rho)

Ep = Ep_kPa*1e3; Epp = Epp_kPa*1e3; % kPa -> Pa

S = hypot(Ep,Epp);

cp = sqrt( 2*(Ep.^2 + Epp.^2) ./ ( 3*rho*(S + Ep) ) );

end

function cp = map_cp_from_G(Gp_Pa, Gpp_Pa, rho)

% Exact loss-aware c_s from measured G', G'' (Pa)

ratio = Gpp_Pa ./ max(Gp_Pa,1e-30);

cp = sqrt( 2*(Gp_Pa.^2 + Gpp_Pa.^2) ./ ( rho .* Gp_Pa .* (1 + sqrt(1 + ratio.^2)) ) );

end

function y = rmse(a,b), y = sqrt(mean((a-b).^2)); end
