# Supplementary material for: Establishing an Elastography calibration standard: Validation of a shear wave TOF device for measuring Elasticity and Viscosity in tissue-mimicking phantoms using rheometry
Source: PLoS One. 2025 Nov 13;20(11):e0335645. doi: 10.1371/journal.pone.0335645 (PMC12614516; doi:10.1371/journal.pone.0335645)
Supplement: S2 File — (ZIP) [file pone.0335645.s002.zip › Dispersive_fits_for_both_Hard_soft_rheometer.docx]

%% KVFD fit with full Q-form and weighted least squares (HARD tissue example)

clear; clc; close all;

%% ----------------------- Input Data — HARD tissue ------------------------

freq = [0.1, 0.125, 0.158, 0.2, 0.251, 0.316, 0.398, 0.5, 0.63, 0.79]'; % Hz

v_mean = [2.05, 2.08, 2.10, 2.13, 2.143, 2.15, 2.16, 2.18, 2.20, 2.19]'; % m/s

v_sem = [0.000, 0.003, 0.015, 0.017, 0.017, 0.011, 0.012, 0.013, 0.011, 0.012]';

% ----------------------- (Optional) SOFT tissue data ----------------------

% freq = [0.1, 0.125893, 0.158489, 0.199526, 0.251189, 0.316228, 0.398107, 0.501187, 0.630957]';

% v_mean = [1.3324, 1.3463, 1.3629, 1.3806, 1.4000, 1.4077, 1.4219, 1.4330, 1.4339]';

% v_sem = [0.0062, 0.00712, 0.00705, 0.00713, 0.00749, 0.00757, 0.00766, 0.00791, 0.00740]';

rho = 1000; % kg/m^3

%% ----------------------- KVFD model (your exact form) --------------------

% Q(p,f) = E0^2 + 2*E0*eta*cos(pi*alpha/2)*(2*pi*f)^alpha + [eta*(2*pi*f)^alpha]^2

Q = @(p,ff) ( p(1).^2 ...

+ 2*p(1).*p(2).*cos(pi*p(3)/2).*(2*pi*ff).^p(3) ...

+ (p(2).^2).*(2*pi*ff).^(2*p(3)) );

% Cs(f) = sqrt( 2*Q / ( 3*rho * ( sqrt(Q) + E0 + eta*(2*pi*f)^alpha*cos(pi*alpha/2) ) ) )

kvfd = @(p,ff) sqrt( 2.*Q(p,ff) ./ ( 3*rho .* ( sqrt(Q(p,ff)) ...

+ p(1) + p(2).*cos(pi*p(3)/2).*(2*pi*ff).^p(3) ) ) );

%% ----------------------- Weighted least squares setup --------------------

% Replace zero SEM with a small positive value to avoid infinite weights

if any(v_sem<=0)

nz = v_sem(v_sem>0);

tiny = max(1e-6, 0.1*min(nz)); % conservative small sigma

v_sem(v_sem<=0) = tiny;

end

W = 1./(v_sem.^2); % inverse-variance weights

sqrtW = sqrt(W);

% Weighted model/target for lsqcurvefit trick

model_w = @(p,ff) kvfd(p,ff(:)).*sqrtW;

y_w = v_mean.*sqrtW;

%% ----------------------- Fit settings -----------------------------------

p0 = [4.17234, 60.543347, 0.5]; % [E0 (Pa), eta (Pa*s^alpha), alpha]

lb = [ 0, 0, 0.13];

ub = [Inf, Inf, 0.99];

opts = optimoptions('lsqcurvefit','Display','off','MaxIterations',2000);

try, opts = optimoptions(opts,'MaxFunctionEvaluations',1e5); catch, end

%% ----------------------- Perform fit (WLS) -------------------------------

[pf,resnorm,~,exitflag,output,~,Jw] = lsqcurvefit(model_w, p0, freq, y_w, lb, ub, opts);

E0 = pf(1); eta = pf(2); alpha = pf(3);

%% ----------------------- Metrics (unweighted residuals) ------------------

v_pred = kvfd(pf, freq);

SS_res = sum((v_mean - v_pred).^2);

SS_tot = sum((v_mean - mean(v_mean)).^2);

R2 = 1 - SS_res/SS_tot;

RMSE = sqrt(mean((v_mean - v_pred).^2));

MAE = mean(abs(v_mean - v_pred));

%% ----------------------- Print results ----------------------------------

fprintf('\n--- KVFD (full Q-form) Weighted Fit ---\n');

fprintf('E0 = %.3e Pa\n', E0);

fprintf('eta = %.3e Pa·s^alpha\n', eta);

fprintf('alpha = %.3f\n', alpha);

fprintf('R^2 = %.4f\n', R2);

fprintf('RMSE = %.4f m/s\n', RMSE);

fprintf('MAE = %.4f m/s\n', MAE);

fprintf('exitflag=%d (lsqcurvefit); iterations=%d\n', exitflag, output.iterations);

%% ----------------------- Publication-quality plot -----------------------

fig = figure('Units','inches','Position',[1 1 7 5], 'Color','w'); hold on;

% Data + error bars

errorbar(freq, v_mean, v_sem, 'o', ...

'MarkerEdgeColor','k','MarkerFaceColor','k','CapSize',8,'LineWidth',1.2, ...

'DisplayName','Measured \pm SE');

% Fitted curve (extend to 1 Hz)

f_fine = linspace(min(freq), 1, 400);

plot(f_fine, kvfd(pf,f_fine), '-', 'LineWidth', 2, 'DisplayName','KVFD fit');

% Axes / labels

xlabel('Frequency (Hz)', 'FontSize',14,'FontWeight','bold');

ylabel('Shear-wave speed (m/s)', 'FontSize',14,'FontWeight','bold');

set(gca,'FontName','Arial','FontSize',12,'LineWidth',1.2);

xlim([min(freq) 1]); grid on; box on;

legend('Location','northwest','Box','off');

% Annotation

txt = {

sprintf('E_0 = %.2e Pa', E0)

sprintf('\\eta = %.2e Pa·s^{\\alpha}', eta)

sprintf('\\alpha = %.3f', alpha)

sprintf('R^2 = %.4f', R2)

sprintf('RMSE = %.4f m/s', RMSE)

sprintf('MAE = %.4f m/s', MAE)

};

annotation('textbox',[0.60,0.15,0.33,0.3],'String',txt, ...

'FitBoxToText','on','BackgroundColor','w','EdgeColor','k','FontSize',11);

%% ----------------------- Exports ----------------------------------------

exportgraphics(fig,'KVFD_Fit_fullQ_to1Hz.png','Resolution',600);

exportgraphics(fig,'KVFD_Fit_fullQ_to1Hz.eps','ContentType','vector');

%% ----------------------- Save parameters --------------------------------

T = table(E0,eta,alpha,R2,RMSE,MAE, ...

'VariableNames',{'E0_Pa','eta_Pa_s_alpha','alpha','R2','RMSE_m_per_s','MAE_m_per_s'});

writetable(T,'KVFD_Fit_fullQ_Results.csv');
