# Supplementary material for: Establishing an Elastography calibration standard: Validation of a shear wave TOF device for measuring Elasticity and Viscosity in tissue-mimicking phantoms using rheometry
Source: PLoS One. 2025 Nov 13;20(11):e0335645. doi: 10.1371/journal.pone.0335645 (PMC12614516; doi:10.1371/journal.pone.0335645)
Supplement: S2 File — (ZIP) [file pone.0335645.s002.zip › E_o_Fixed_reoptimize_eta.docx]

%% constrained_refit_tof_E0_fixed.m (simple, journal-ready + downloads)

% Constrained ToF refit with E0 fixed to the rheometer value (5.49 kPa)

% Re-optimizes {eta, alpha} using weighted least squares on c_p(f)

% Uses exact loss-aware map from (E′,E″) to phase velocity.

% Optional: also compute the unconstrained ToF fit for comparison.

%

% Outputs a clean figure and saves:

% - fig_constrained_refit_cp.pdf (vector)

% - fig_constrained_refit_cp.png (600 dpi)

% - fig_constrained_refit_cp.eps (vector, fallback if needed)

clear; clc; close all;

%% -------------------- Data (ToF) --------------------

rho = 1000; % kg/m^3

E0_fixed_kPa = 5.49; % kPa (fixed to rheometer value)

% Frequencies (Hz) and three repeats per frequency

f_tof = [40 60 80 100 120 140 160 180]';

cp_reps = [ ...

1.360 1.390 1.390; % 40

1.413 1.444 1.458; % 60

1.479 1.497 1.501; % 80

1.492 1.505 1.523; % 100

1.513 1.541 1.608; % 120

1.577 1.603 1.632; % 140

1.660 1.688 1.705; % 160

1.694 1.714 1.750]; % 180

cp_mean = mean(cp_reps,2);

cp_SE = std(cp_reps,0,2) / sqrt(size(cp_reps,2));

w = 1 ./ max(cp_SE, 1e-6); % weights for WLS

%% ----------------- Options & initial guesses -----------------

use_lsq = exist('lsqnonlin','file')==2 && license('test','Optimization_Toolbox');

% Bounds for [eta, alpha]

lb = [1e-8, 1e-3];

ub = [1e3, 0.999];

% Good starting guesses (based on earlier ToF fit)

eta0 = 0.012; % kPa*s^alpha

alpha0 = 0.86;

%% ========== (Optional) Unconstrained ToF fit (E0 free) ==========

do_unconstrained = true;

if do_unconstrained

theta0_unc = [5.0, 0.02, 0.6]; % [E0, eta, alpha] initial guess

lb_unc = [1e-6, 1e-8, 1e-3];

ub_unc = [1e3, 1e3, 0.999];

if use_lsq

opts = optimoptions('lsqnonlin','Display','off','MaxFunctionEvaluations',5e4,'MaxIterations',2e3);

[theta_unc,~,res_unc,~,~,~,J_unc] = lsqnonlin(@(th) res_ToF_E0free(th,f_tof,cp_mean,w,rho), theta0_unc, lb_unc, ub_unc, opts);

CI_unc = paramCI_generic(theta_unc,res_unc,J_unc);

else

obj = @(th) sum(res_ToF_E0free(clip(th,lb_unc,ub_unc),f_tof,cp_mean,w,rho).^2);

theta_unc = clip(fminsearch(obj, theta0_unc, optimset('Display','off')), lb_unc, ub_unc);

CI_unc = nan(3,2);

end

E0_unc = theta_unc(1);

eta_unc = theta_unc(2);

alp_unc = theta_unc(3);

[Ep_unc,Epp_unc] = kvfd_E(f_tof, E0_unc, eta_unc, alp_unc);

cp_fit_unc = map_cp(Ep_unc,Epp_unc,rho);

RMSE_unc = rmse(cp_fit_unc, cp_mean);

MAPE_unc = 100*mean(abs(cp_fit_unc - cp_mean)./cp_mean);

end

%% ======= Constrained ToF refit (E0 fixed = 5.49 kPa) =======

x0 = [eta0, alpha0]; % [eta, alpha]

if use_lsq

opts = optimoptions('lsqnonlin','Display','off','MaxFunctionEvaluations',5e4,'MaxIterations',2e3);

[x_opt,~,res_fix,~,~,~,J_fix] = lsqnonlin(@(x) res_ToF_E0fixed(x,f_tof,cp_mean,w,rho,E0_fixed_kPa), x0, lb, ub, opts);

CI_fix = paramCI_generic(x_opt,res_fix,J_fix); % 2x2 CI for [eta, alpha]

else

obj = @(x) sum(res_ToF_E0fixed(clip(x,lb,ub),f_tof,cp_mean,w,rho,E0_fixed_kPa).^2);

x_opt = clip(fminsearch(obj, x0, optimset('Display','off')), lb, ub);

CI_fix = nan(2,2);

end

eta_fix = x_opt(1);

alp_fix = x_opt(2);

[Ep_fix, Epp_fix] = kvfd_E(f_tof, E0_fixed_kPa, eta_fix, alp_fix);

cp_fit_fix = map_cp(Ep_fix, Epp_fix, rho);

RMSE_fix = rmse(cp_fit_fix, cp_mean);

MAPE_fix = 100*mean(abs(cp_fit_fix - cp_mean)./cp_mean);

%% -------------------- Print summary --------------------

fprintf('\n=== Constrained ToF refit (E0 fixed to 5.49 kPa) ===\n');

fprintf('eta = %.6g kPa·s^alpha', eta_fix);

if all(~isnan(CI_fix(1,:)))

fprintf(' (95%% CI [%.6g, %.6g])', CI_fix(1,1), CI_fix(1,2));

end

fprintf('\nalpha= %.6f', alp_fix);

if all(~isnan(CI_fix(2,:)))

fprintf(' (95%% CI [%.6f, %.6f])', CI_fix(2,1), CI_fix(2,2));

end

fprintf('\nRMSE(c_p) = %.4f m/s | MAPE(c_p) = %.2f %%\n', RMSE_fix, MAPE_fix);

if do_unconstrained

fprintf('\n--- Unconstrained ToF (for comparison) ---\n');

fprintf('E0 = %.4f kPa', E0_unc);

if all(~isnan(CI_unc(1,:))), fprintf(' (95%% CI [%.4f, %.4f])', CI_unc(1,1), CI_unc(1,2)); end

fprintf('\neta = %.6g kPa·s^alpha', eta_unc);

if all(~isnan(CI_unc(2,:))), fprintf(' (95%% CI [%.6g, %.6g])', CI_unc(2,1), CI_unc(2,2)); end

fprintf('\nalpha= %.6f', alp_unc);

if all(~isnan(CI_unc(3,:))), fprintf(' (95%% CI [%.6f, %.6f])', CI_unc(3,1), CI_unc(3,2)); end

fprintf('\nRMSE(c_p) = %.4f m/s | MAPE(c_p) = %.2f %%\n', RMSE_unc, MAPE_unc);

end

%% ----------------------- Plot (journal-ready, simple) ----------------

set(0,'DefaultFigureRenderer','painters'); % vector output

fig = figure('Color','w','Units','centimeters','Position',[2 2 8.5 6.5]); % ~single column

hold on; grid on; box on;

% Data with SE bars

errorbar(f_tof, cp_mean, cp_SE, 'o', 'Color','k','MarkerFaceColor','w', ...

'MarkerSize',4,'CapSize',6,'LineStyle','none', 'DisplayName','TOF mean ± SE');

% Constrained fit

plot(f_tof, cp_fit_fix, '-', 'LineWidth',1.8,'Color',[0.00 0.45 0.74], ...

'DisplayName','KVFD fit (E\_0 fixed = 5.49 kPa)');

% (Optional) Unconstrained fit for comparison

if do_unconstrained

plot(f_tof, cp_fit_unc, '--', 'LineWidth',1.8,'Color',[0.85 0.33 0.10], ...

'DisplayName','KVFD fit (unconstrained)');

end

xlabel('Frequency (Hz)');

ylabel('Shear wave speed (m/s)');

xlim([min(f_tof)-5, max(f_tof)+5]);

ylim([min([cp_mean;cp_fit_fix])-0.05, max([cp_mean;cp_fit_fix])+0.05]);

legend('Location','northwest','Box','off');

set(gca,'TickDir','out','LineWidth',0.8,'FontName','Times','FontSize',8);

ax = gca; ax.XMinorTick='on'; ax.YMinorTick='on';

%% ----------------------- Save (vector + 600 dpi) -------------------

outBase = 'fig_constrained_refit_cp';

if exist('exportgraphics','file')==2

exportgraphics(fig, [outBase '.pdf'], 'ContentType','vector'); % vector

exportgraphics(fig, [outBase '.png'], 'Resolution', 600); % 600 dpi

% Try EPS for journals that prefer it

try

exportgraphics(fig, [outBase '.eps'], 'ContentType','vector');

catch

print(fig, [outBase '.eps'], '-depsc','-painters');

end

else

% Fallback for older MATLAB

print(fig, [outBase '.pdf'], '-dpdf','-painters');

print(fig, [outBase '.png'], '-dpng','-r600');

print(fig, [outBase '.eps'], '-depsc','-painters');

end

fprintf('\nSaved: %s.pdf (vector), %s.png (600 dpi), and %s.eps (vector)\n', outBase, outBase, outBase);

%% ====================== Helpers =======================

function r = res_ToF_E0fixed(x,f_Hz,cp_mean,w,rho,E0_kPa)

% x = [eta_kPa_s_alpha, alpha]

[Ep,Epp] = kvfd_E(f_Hz, E0_kPa, x(1), x(2));

cp_hat = map_cp(Ep,Epp,rho);

r = (cp_hat - cp_mean) .* w;

end

function r = res_ToF_E0free(theta,f_Hz,cp_mean,w,rho)

% theta = [E0_kPa, eta_kPa_s_alpha, alpha]

[Ep,Epp] = kvfd_E(f_Hz, theta(1), theta(2), theta(3));

cp_hat = map_cp(Ep,Epp,rho);

r = (cp_hat - cp_mean) .* w;

end

function [Ep, Epp] = kvfd_E(f_Hz, E0_kPa, eta_kPa_s_a, alpha)

w = 2*pi*f_Hz(:);

pow = w.^alpha;

c = cos(pi*alpha/2); s = sin(pi*alpha/2);

Ep = E0_kPa + eta_kPa_s_a .* pow .* c; % kPa

Epp = eta_kPa_s_a .* pow .* s; % kPa

end

function cp = map_cp(Ep_kPa, Epp_kPa, rho)

% Exact loss-aware phase velocity from (E′,E″) (kPa) → c_p (m/s)

Ep = Ep_kPa*1e3; Epp = Epp_kPa*1e3; % Pa

S = hypot(Ep,Epp);

cp = sqrt( 2*(Ep.^2 + Epp.^2) ./ ( 3*rho*(S + Ep) ) );

end

function y = rmse(a,b), y = sqrt(mean((a-b).^2)); end

function th = clip(th,lb,ub), th = max(min(th,ub),lb); end

function CI = paramCI_generic(theta,res,J)

% 95% CI from Gauss–Newton (handles sparse J from lsqnonlin)

dof = max(numel(res) - numel(theta), 1);

s2 = max(sum(res.^2)/dof, eps);

J = full(J);

F = J.'*J;

n = size(F,1);

if rcond(F) < 1e-12

lam = 1e-6 * trace(F) / n;

F = F + lam*eye(n);

end

[R,p] = chol(F);

if p==0

Cov = s2 * (R \ (R' \ eye(n)));

else

Cov = s2 * pinv(F);

end

se = sqrt(diag(Cov));

if exist('tinv','file')==2, z = tinv(0.975, dof); else, z = 1.96; end

CI = [theta(:)-z*se(:), theta(:)+z*se(:)];

end
