# Supplementary material for: Establishing an Elastography calibration standard: Validation of a shear wave TOF device for measuring Elasticity and Viscosity in tissue-mimicking phantoms using rheometry
Source: PLoS One. 2025 Nov 13;20(11):e0335645. doi: 10.1371/journal.pone.0335645 (PMC12614516; doi:10.1371/journal.pone.0335645)
Supplement: S2 File — (ZIP) [file pone.0335645.s002.zip › Sweep_plot_for_Hard_phantom1.docx]

%% Publication-ready plot & table (values in Pascal)

clc, clear, close all

%% ------------------------------- 1) DATA (MPa)_hard tissue --------------------------

G1 = [4.22294e-3 4.49074e-3 4.61294e-3 4.77921e-3 4.89258e-3 4.99701e-3 5.08828e-3 5.13134e-3 5.25930e-3 5.27489e-3 5.16288e-3 ...

7.30227e-3 7.63672e-3 7.71185e-3 7.93640e-3 8.12762e-3 8.24126e-3 8.37839e-3 8.44050e-3 8.48482e-3 8.44264e-3 8.11872e-3 3.74892e-3];

G2 = [4.18491e-3 4.27771e-3 4.36009e-3 4.50073e-3 4.52835e-3 4.57606e-3 4.64438e-3 4.69529e-3 4.78208e-3 4.76955e-3 5.83412e-3 ...

6.03247e-3 6.14641e-3 6.11371e-3 6.13423e-3 6.36714e-3 6.41020e-3 6.69807e-3 6.81044e-3 6.88789e-3 6.78531e-3 6.76500e-3 1.69045e-3];

G3 = [4.17234e-3 4.33001e-3 4.46005e-3 4.60506e-3 4.67485e-3 4.81322e-3 4.90098e-3 4.90473e-3 4.98797e-3 4.99845e-3 5.02241e-3 ...

7.33589e-3 7.42248e-3 7.11865e-3 6.81301e-3 6.95114e-3 7.10091e-3 7.28291e-3 7.37591e-3 7.36435e-3 7.48101e-3 7.82249e-3 2.93921e-3];

L1 = [6.72776e-4 6.71993e-4 6.51606e-4 6.57551e-4 6.76998e-4 6.62017e-4 6.57698e-4 7.28244e-4 7.33569e-4 7.23193e-4 6.38051e-4 ...

8.50678e-4 1.04553e-3 1.20431e-3 1.06723e-3 9.09692e-4 8.78763e-4 7.64264e-4 7.26017e-4 7.63024e-4 7.48946e-4 1.13422e-3 1.52560e-3];

L2 = [5.29982e-4 5.55519e-4 4.98910e-4 4.98436e-4 5.42853e-4 5.57524e-4 5.53708e-4 5.87188e-4 6.23561e-4 6.06911e-4 7.74414e-4 ...

7.79810e-4 9.77584e-4 1.01666e-3 9.60157e-4 9.06311e-4 8.03310e-4 7.14919e-4 6.23900e-4 6.60162e-4 7.32878e-4 1.02536e-3 1.36663e-3];

L3 = [5.43347e-4 5.85985e-4 5.62423e-4 6.06348e-4 6.13348e-4 6.00220e-4 6.32443e-4 6.45922e-4 6.73723e-4 6.99523e-4 7.11744e-4 ...

9.72964e-4 1.03220e-3 1.05677e-3 9.92322e-4 9.14172e-4 8.43980e-4 7.78573e-4 7.05489e-4 7.37810e-4 8.24962e-4 1.26875e-3 1.54993e-3];

freq = [0.10000000 0.12589300 0.15848900 0.19952600 0.25118900 0.31622800 0.39810700 0.50118700 0.63095700 0.79432800 ...

1.00000000 1.25893000 1.58489000 1.99526000 2.51189000 3.16228000 3.98107000 5.01187000 6.30957000 7.94328000 ...

10.00000000 12.58930000 15.84890000];

%% -------------------------- 2) UNIT CONVERSION --------------------------

MPa2Pa = 1e6;

G1 = G1*MPa2Pa; G2 = G2*MPa2Pa; G3 = G3*MPa2Pa;

L1 = L1*MPa2Pa; L2 = L2*MPa2Pa; L3 = L3*MPa2Pa;

%% ---------------------- 3) MEAN & STANDARD ERROR ------------------------

G = [G1; G2; G3]; L = [L1; L2; L3];

N = size(G,1);

Gm = mean(G,1); Gs = std(G,0,1)/sqrt(N);

Lm = mean(L,1); Ls = std(L,0,1)/sqrt(N);

%% only 0.1 – 0.794 Hz

keep = freq <= 0.79432800;

f_sub = freq(keep);

Gm_s = Gm(keep); Gs_s = Gs(keep);

Lm_s = Lm(keep); Ls_s = Ls(keep);

% ensure positive error-bar lows for log scale

Gs_s = min(Gs_s, 0.99*Gm_s);

Ls_s = min(Ls_s, 0.99*Lm_s);

%% ---------------- 4) OUTPUT FOLDER & TIMESTAMP --------------------------

ts = datestr(now,'yyyymmdd_HHMMSS');

outdir = fullfile(pwd,['PublicationPlots_' ts]);

if ~exist(outdir,'dir'), mkdir(outdir), end

%% ----------------------------- 5) PLOT ----------------------------------

fig = figure('Units','inches','Position',[1 1 6.4 4],'Color','w');

hold on

h1 = errorbar(f_sub,Gm_s,Gs_s,'-s','LineWidth',2,'MarkerSize',5,...

'Color',[0 0.25 0.8],'MarkerFaceColor',[0 0.25 0.8],...

'DisplayName',"G' (mean ± SE)");

h2 = errorbar(f_sub,Lm_s,Ls_s,'--s','LineWidth',2,'MarkerSize',5,...

'Color',[0.85 0.33 0.1],'MarkerFaceColor',[0.85 0.33 0.1],...

'DisplayName',"G'' (mean ± SE)");

ax = gca;

ax.FontName = 'Arial';

ax.FontSize = 14;

ax.LineWidth = 1.2;

ax.XScale = 'log';

ax.YScale = 'log';

ax.YMinorTick = 'off';

ax.TickLabelInterpreter = 'tex';

xlabel('Frequency (Hz)','FontWeight','bold')

ylabel('Storage and loss modulus (Pa)','FontWeight','bold')

grid on; box on

% robust y-limits with headroom so legend won't overlap curves

ymin = min([Gm_s-Gs_s, Lm_s-Ls_s],[],'all');

ymax = max([Gm_s+Gs_s, Lm_s+Ls_s],[],'all');

ymin = max(ymin, eps);

ymin = 10^floor(log10(ymin));

ymax = 10^ceil(log10(ymax)); % go to next decade (gives generous top gap)

ylim([ymin ymax])

yticks(10.^(floor(log10(ymin)):ceil(log10(ymax))))

xlim([0.08 1.5])

% your requested legend placement and styling

legend('Location','southeast','Box','off');

% small tweaks to keep it compact

lgd = legend;

lgd.FontSize = 12;

lgd.ItemTokenSize = [20 8];

%% ----------------------------- 6) SAVE ----------------------------------

base = fullfile(outdir,['Gmodulus_0p1-1p3Hz_' ts]);

set(fig,'Renderer','painters')

try

print(fig,[base '.eps'],'-depsc','-painters','-r600')

catch ME

warning('EPS export failed:\n%s\nPDF & PNG were still written.',ME.message)

end

exportgraphics(fig,[base '.pdf'],'ContentType','vector')

exportgraphics(fig,[base '.png'],'Resolution',600)

%% ----------------------- 7) TABLE (in Pa) -------------------------------

makefmt = @(m,se) sprintf('%.2f ± %.2f',m,se);

Gfmt = arrayfun(makefmt,Gm_s,Gs_s,'uniform',false);

Lfmt = arrayfun(makefmt,Lm_s,Ls_s,'uniform',false);

T = table(f_sub', Gm_s', Gs_s', Lm_s', Ls_s', Gfmt', Lfmt', ...

'VariableNames',{'Frequency_Hz','Gprime_Pa','SE_Gprime','Gdprime_Pa','SE_Gdprime','Gprime_fmt','Gdprime_fmt'});

disp(T)

writetable(T,[base '_table.csv'])

writetable(T,[base '_table.xlsx'])

fprintf('\nAll files saved in:\n%s\n',outdir)

if ispc, winopen(outdir)

elseif ismac, system(['open "',outdir,'"'])

elseif isunix,system(['xdg-open "',outdir,'"'])

end
